# Exploring the versatility of *Drosophila melanogaster* as a model organism in biomedical research: a comprehensive review

**DOI:** 10.1080/19336934.2024.2420453

**Published:** 2024-12-25

**Authors:** Ayomide Victor Atoki, Patrick Maduabuchi Aja, Tijjani Salihu Shinkafi, Erick Nyakundi Ondari, Adekunle Ismahil Adeniyi, Ilemobayo Victor Fasogbon, Reuben Samson Dangana, Umar Uthman Shehu, Akinpelumi Akin-Adewumi

**Affiliations:** a Department of Biochemistry, Kampala International University, Ishaka, Uganda; b Department of Biochemistry, Faculty of Science, Ebonyi State University, Abakaliki, Nigeria; c School of Pure and Applied Sciences, Department of Biological Sciences, Kisii University, Kisii, Kenya; d Department of Physiology, Kampala International University, Ishaka, Uganda; e College of Medicine, Faculty of Dentistry, University of Ibadan, Ibadan, Nigeria

**Keywords:** Drosophila melanogaster, disease modelling, neurodegeneration, cancer, metabolic diseases, genetic tools, cardiac diseases, muscular diseases

## Abstract

*Drosophila melanogaster* is a highly versatile model organism that has profoundly advanced our understanding of human diseases. With more than 60% of its genes having human homologs, *Drosophila* provides an invaluable system for modelling a wide range of pathologies, including neurodegenerative disorders, cancer, metabolic diseases, as well as cardiac and muscular conditions. This review highlights key developments in utilizing *Drosophila* for disease modelling, emphasizing the genetic tools that have transformed research in this field. Technologies such as the GAL4/UAS system, RNA interference (RNAi) and CRISPR-Cas9 have enabled precise genetic manipulation, with CRISPR-Cas9 allowing for the introduction of human disease mutations into orthologous *Drosophila* genes. These approaches have yielded critical insights into disease mechanisms, identified novel therapeutic targets and facilitated both drug screening and toxicological studies. Articles were selected based on their relevance, impact and contribution to the field, with a particular focus on studies offering innovative perspectives on disease mechanisms or therapeutic strategies. Our findings emphasize the central role of *Drosophila* in studying complex human diseases, underscoring its genetic similarities to humans and its effectiveness in modelling conditions such as Alzheimer’s disease, Parkinson’s disease and cancer. This review reaffirms *Drosophila*’s critical role as a model organism, highlighting its potential to drive future research and therapeutic advancements.

## Introduction

*Drosophila* is a genus of flies that is part of the *Drosophilidae* family. The members within this family are often colloquially dubbed ‘small fruit flies’, occasionally referred to as pomace flies, vinegar flies, or wine flies, as they are commonly found hovering around decaying or overly ripe fruit. They are quite different from the *Tephritidae*, a related family of insects often known as fruit flies (also termed ‘real fruit flies’); tephritids feed predominantly on ripe or unripe fruit, and many species, particularly the Mediterranean fruit fly, are considered harmful agricultural pests. Since its introduction more than a century ago, in particular, *D. melanogaster*, among the myriad species of *Drosophila*, has been extensively employed in genetic studies and serves as a prominent model organism in biomedical research and in the field of developmental biology, notably in genetics and molecular biology [1]. In modern biological literature, the terms ‘fruit fly’ and ‘*Drosophila*’ are often used interchangeably with *Drosophila melanogaster*. Nonetheless, the genus encompasses over one thousand five hundred species, exhibiting considerable diversity in behaviour and appearance, as well as preferred breeding environments [2]. More specifically, more than 65–70% of the genes responsible for human disease have been found in *D. melanogaster* [3,4], making it an effective model organism for research in the domains of biochemistry, molecular biology, genetics, and cell biology. *Drosophila* presents comparative advantages over other models for biological investigation due to its rapid generation turnover, short life cycle, and ease of handling and maintenance in the laboratory, allowing for large-scale studies [5].

### Selection criteria and focus areas

This review is based on a comprehensive search of the literature across several databases, including Scopus, Science Direct, Google Scholar and PubMed, using keywords such as ‘*Drosophila*’, *‘Drosophila* genes human homologs’, *‘Drosophila* in neuronal biology’, ‘conserved genes in *Drosophila*’, *‘Drosophila* in toxicology’, *‘Drosophila* in cardiac disease’, *‘Drosophila* in muscular conditions’, *‘Drosophila* in infectious diseases’ and *‘Drosophila* in cancer research’. Given the vast number of publications in the field, the review focuses on diseases with the highest research output, particularly those that have led to substantial breakthroughs in understanding disease mechanisms or treatment strategies using *Drosophila* models.

The articles discussed were selected based on their relevance, impact and contribution to the field. Priority was given to studies that introduced new models, provided significant insights into disease mechanisms, or demonstrated novel therapeutic approaches. While the review predominantly covers neurological diseases, where *Drosophila* has been extensively used, other areas such as cardiac and muscular diseases are mentioned to provide a broader context.

## Habitat

The tropics harbour the highest diversity of *Drosophila* species, with numerous distinct variations existing within this genus. In the Hawaiian Islands, *Drosophila* spread and produced over 800 different species [6]. They can be found across a range of habitats including deserts, alpine regions, urban areas, wetlands, and tropical rainforests. Additionally, some species in northern regions undergo hibernation. The *Drosophila montana*, which is a northern species are primarily found at high altitudes [7], is the best at withstanding cold [8]. Most species of *Drosophila* reproduce in various forms of decomposed fungal and plant matter, including flowers, bark, mushrooms, and ripe fruit. One species’ larvae, *D. suzukii*, can occasionally be a problem and feed on fresh fruit as well [9]. A few creatures have evolved into predators or parasites. While certain species can be drawn to baits made from fermenting mushrooms or bananas, other species are not drawn to any sort of bait. Males may gather in leks, performing courtship away from breeding grounds, or they may assemble around an ideal breeding material where they compete for female flies [10].

Many species of *Drosophila*, especially the *melanogasters*, the *simulans* and also the *immigrans* are sometimes referred to as domestic species because of their intimate relationship with human being. These species, along with others from the similar genus *Zaprionus indianus*, have unintentionally spread over the world as a result of human activities like fruit shipments [11,12].

## Reproduction

*D*. *bifurca* has been shown to be the organism with the longest sperm cell on earth, with a length of 58 mm (2.3 Inches) long [13]. The cells are transferred into female flies in the form of tangled coils and generally have a long, thread-like tail. There aren’t many gigantic sperm cells produced by the other *Drosophila* species, with *D. bifurca’s* being the longest [14]. Sperm cells from *D. melanogaster* are relatively moderate in length, which is about 1.8 mm long, however they are still roughly thirty-five times lengthier than sperm from a human being. It has been shown that a number of *D. melanogaster* species mate through traumatic insemination [15].

### Life cycle of the fruit fly

**Figure 1.**
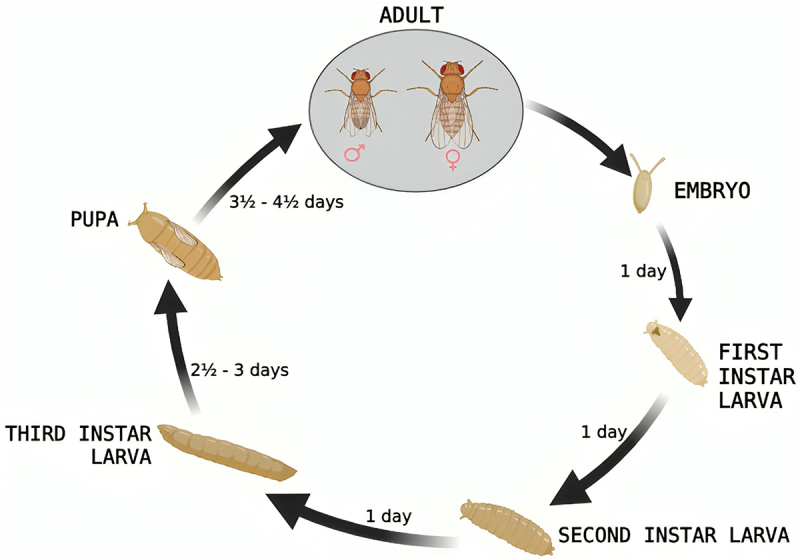
Life cycle of *Drosophila melanogaster.*

*Drosophila* has a four-step life cycle, similar to butterflies and moths: egg, larva, pupa, and fly. The embryo grows in the egg for about a day (at 25 °C) after fertilization before being released as a larva (Figure 1). During a span of five days, the larva of *Drosophila* consumes food, undergoes growth, and experiences three moulting stages before entering the pupal stage. Subsequently, it undergoes a four-day metamorphosis process, culminating in the emergence of the adult fly. During metamorphosis, the embryonic and larval tissues are removed. The ‘imaginal discs’, clusters of cells vital for early embryonic development, serve as the precursors for adult tissues, including legs, wings, and eyes, in *Drosophila*. In *Drosophila*, mature tissues typically do not regenerate. For instance, if a fly’s wings are severed, they will not regenerate, similar to the case in humans. In recent years, imaginal discs have offered a priceless model system for studying the genetics of tissue regeneration because they do have the ability to repair if damaged in specific circumstances [15].

The ability to reproduce varies greatly amongst *Drosophila* species. Some species, like *D. melanogaster*, reproduce in huge, comparatively uncommon materials and possess ovaries that have the ability of releasing 10–20 mature eggs at a time, allowing them to be laid all at once at the same location. Some species, such as those that reproduce on more plentiful but less nutrient-rich substrates like leaves, might lay just a single egg per day. Near the front end of the eggs are respiratory filaments (which can be one or more than one); protrude tips, which facilitate oxygen access to the embryo. Larvae consume the yeasts and bacteria that are present on the breeding substrate that is decomposing rather than the actual vegetable matter. The length of development varies significantly among flies’ species (usually, between seven and sixty days; or sometimes, more than sixty days) and is influenced by elements like temperature, substrate on which breeding is taking place, and population density.

Environmental cycles impact egg-laying in fruit flies. Larvae are produced in greater quantities by eggs laid during periods (such as night) when the possibility of survival is higher than by eggs laid during the day. Given that this behaviour provides a significant reproductive benefit, *D. melanogaster* would adjust to environmental cycles as a result of the disparity in reproductive success [16]. Their average life expectancy is 35 to 45 days [17].

### Mating systems

#### Courtship behaviour

Male *Drosophila’s* courtship activity is a desirable behaviour [18]. Females react based on how they interpret the male’s behaviour [19]. *Drosophila* males and females utilizes a number of corporeal signals to initiate and evaluate a possible mate’s courtship readiness [18,20]. The behaviours that serve as signals are the following: pheromone emission, positioning, spreading of wings, production of sounds by tapping the legs, production of vibration through wing flapping, stomach bending, and actually engaging in copulation [18,21]. Quite a number of studies have been conducted on the songs of *Drosophila simulans* and *Drosophila melanogaster*; the sinusoidal nature of these luring tunes vary between species [20].

Genes that encode certain sex-related phenomena, which have been linked to courtship behaviour in males and in females, have also been evaluated for courtship behaviour in *Drosophila melanogaster* [18]. A collection of genes connected to sex behaviour known as fruitless (fru) and doublesex (dsx) have been the subject of recent studies [18,22]. In fruit flies, the fruitless (fru) gene plays a role in the network that controls male courtship behaviour; when this gene is mutated, altered same-sex sexual behaviour in males is seen [23]. The fru mutation causes male *Drosophila* to focus their courtship on other men rather than on females as it would normally [24]. Loss of mutation of fru gene resulted in the return of the standard courting behaviour [24].

#### Polyandry

Among *Drosophila*, a very common system of mating is polyandry; female flies mate with numerous males, has proven a successful mating tactic for fruit fly [25–27]. Pre-copulatory and post-copulatory mating have advantages. Pre-copulatory techniques refer to the mate-selection behaviours and genetic contributions, such as the creation of gametes, that are displayed by male and in female flies [27]. Sperm competition, the frequency of mating, and meiotic drive based on sex ratio are post-copulatory strategies [25,27].

The number of mating partners in North American *Drosophila pseudoobscura* polyandry varies [25]. Chromosomal variations of the third chromosome and the frequency of female mating are correlated [25]. Re-mating by females is thought to occur because of the inverted polymorphism [25]. The sex-ratio meiotic desire may have a role in the stability of these polymorphisms [26]. However, the primary mating method for *Drosophila subobscura* is monandry, which is unusual for *Drosophila* [28].

#### Sperm competition

Polyandrous *Drosophila* females employ the process of sperm competition to improve the fitness of their progeny [29,30]. The spermathecae and seminal receptacle, two sperm storage organs in the female *Drosophila*, help her to select the sperm that will fertilize her eggs [29]. But some *Drosophila* species have evolved to only employ one or the other [31]. When it comes to mysterious feminine choice, females have little control [32]. Using cryptic choice, which is one of the numerous mechanisms of post-copulation, female *Drosophila* can identify and expel sperm, which lowers the likelihood of inbreeding [33]. According to Manier et al. [30], insemination, storage of sperm, and fertilization of sperm are the three steps post-copulatory sexual selection of *D. melanogaster, D. mauritiana and D. simulans* are divided into [30]. There are differences between each stage of the aforementioned species that contribute to natural selection [30]. According to research by Lüpold *et al*. [34] and Zajitschek *et al*. [35], this sperm rivalry was a major factor in the formation of reproductive isolation throughout speciation.

## Drosophila culture

**Figure 2.**
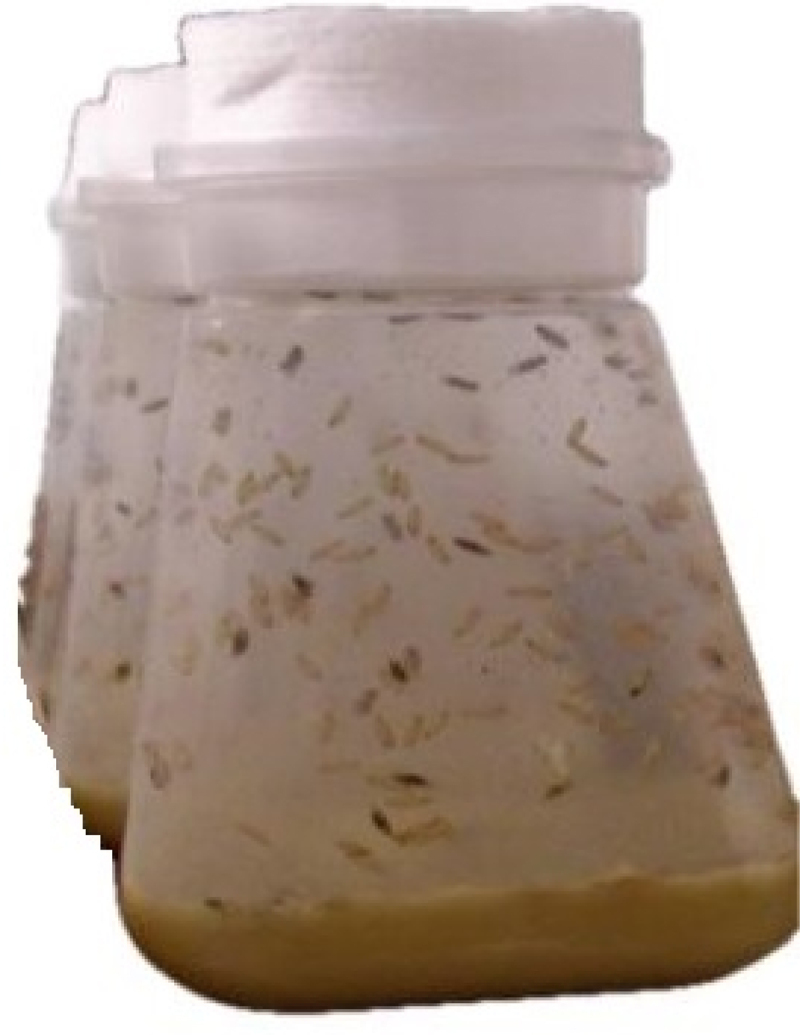
*Drosophila* culture.

*Drosophila* is generally affordable and simple to maintain; in fact, they are frequently used as an instruction tool in high school biology classes to illustrate the fundamental concepts of genetics and heredity. Furthermore, their utilization in laboratories is generally unrestricted due to the absence of significant ethical and safety concerns. Since a female *Drosophila* can lay up to a hundred egg in a day, for up to 20 days, an embryo develops into a fertile adult fly in about 5–10 days at 25°C [36]. Therefore, if necessary, it is quite simple to produce a huge number of flies for a scientific investigation. In the past, laboratory *Drosophila* were housed in containers with rotting banana pulp [37], but nowadays, it is more customary to culture them in containers with a slurry-like food that is classically created from a combination of water, soy flour, yeast, corn syrup, malt extract, agar and cornmeal [36] as shown in Figure 2. The food must be both firm enough to prevent flies from being stuck in it and soft enough to allow the larvae to burrow through it and feed.

The fundamental recipe can be altered in a variety of ways, and pre-mixed formulations are also offered. Foam or cotton wool plugs are placed into bottles and vials to keep mites and other pests out while also preventing fly egress. If it is required to manipulate individual flies, *Drosophila* can be carefully anesthetized in carbon (IV) dioxide. In the majority of well-known fly laboratories, flies are positioned on absorbent pads attached to a supply of carbon (IV) dioxide, manipulated using a fine-tipped paintbrush, and observed through a stereomicroscope. Carbon (IV) oxide can be substituted with ether, and a stereomicroscope can be replaced with a magnifying glass. Since *Drosophila* gametes or embryos cannot currently be effectively frozen, it is vital to preserve *Drosophila* strains as living stocks. Fly stocks are typically maintained around eighteen-degree centigrade since doing so shortens the life cycle to about twenty-eight days. This implies that each *Drosophila* stock only needs to be fed with fresh food once a month, under certain conditions.

## Genetic tools in Drosophila research

*D*. *melanogaster* has become a powerful model organism in biomedical research largely due to the sophisticated genetic tools available for manipulating its genome. These tools enable researchers to study gene function, model human diseases and explore complex biological processes with precision. This section provides an overview of the most commonly used genetic tools in *Drosophila* research, illustrating how they contribute to the study of human diseases.

### The GAL4/UAS system

One of the most widely used genetic tools in *Drosophila* research is the GAL4/UAS system, which allows for the targeted expression of genes in specific tissues or at particular developmental stages. This system consists of two components: the GAL4 gene, which encodes a yeast transcriptional activator, and the upstream activating sequence (UAS), which is recognized by GAL4. By placing the GAL4 gene under the control of a tissue-specific promoter and the gene of interest under the control of a UAS element, researchers can drive the expression of the target gene in specific tissues or cells [38].

The GAL4/UAS system is incredibly versatile and has been used to model a wide range of human diseases in *Drosophila*. For instance, it allows for the expression of human disease-related genes, such as amyloid-beta or alpha-synuclein, in specific nurons to study neurodegenerative diseases like Alzheimer’s and Parkinson’s disease [39]. It can also be used to knock down gene expression through RNA interference (RNAi) by driving the expression of double-stranded RNA (dsRNA) that targets specific genes for silencing.

### RNA interference (RNAi)

RNA interference (RNAi) is a powerful technique for gene silencing in *Drosophila*. RNAi involves the introduction of double-stranded RNA (dsRNA) that is complementary to the mRNA of the target gene. The dsRNA is processed by the RNA-induced silencing complex (RISC), which degrades the target mRNA, leading to a reduction in gene expression [40].

In *Drosophila*, RNAi can be used in a tissue-specific manner by combining it with the GAL4/UAS system. This allows researchers to knock down genes in specific tissues or at specific times during development, making it a valuable tool for studying gene function and modelling diseases. For example, RNAi has been used to silence genes involved in insulin signalling, allowing researchers to study the effects on metabolism and diabetes [41].

### CRISPR-cas9 genome editing

The CRISPR-Cas9 system has revolutionized genetic research by allowing precise and efficient genome editing. CRISPR-Cas9 uses a guide RNA (gRNA) to target specific DNA sequences, and the Cas9 enzyme introduces double-strand breaks at the target site. This can result in gene knockouts, insertions, or replacements through homologous recombination or non-homologous end joining [42]. In *Drosophila*, CRISPR-Cas9 has been used to generate mutants for studying gene function, create disease models, and investigate genetic interactions. This tool is particularly valuable for creating precise genetic modifications, such as introducing specific mutations that are known to cause human diseases. For example, CRISPR-Cas9 has been used to create *Drosophila* models of cancer by introducing mutations in tumour suppressor genes or oncogenes [43]. A more comprehensive discussion on the applications and advancements of CRISPR-Cas9 in Drosophila research is provided in a dedicated section later in the manuscript.

### FLP/FRT system for mitotic recombination

The FLP/FRT system is another genetic tool used in *Drosophila* research for generating mosaic animals, where only specific cells or tissues are genetically altered. This system is based on the site-specific recombination of DNA sequences known as FRT sites by the FLP recombinase, an enzyme derived from yeast [44]. When FRT sites are placed on homologous chromosomes, FLP recombinase can induce recombination between them, resulting in genetic mosaics.

The FLP/FRT system is particularly useful for studying gene function in a tissue-specific manner and for modelling diseases like cancer. By creating clones of cells with specific genetic alterations, researchers can study how these mutations contribute to tumorigenesis or other disease processes without affecting the entire organism.

### MARCM (Mosaic analysis with a repressible cell marker)

Mosaic Analysis with a Repressible Cell Marker (MARCM) is a technique that combines the GAL4/UAS system with the FLP/FRT system to create genetically distinct clones of cells in a background of wild-type tissue. This is achieved by using a cell marker, such as GFP, that is repressed in wild-type cells but expressed in mutant clones [45].

MARCM is particularly useful for studying the cell-autonomous effects of gene mutations, allowing researchers to examine how specific genetic alterations affect cell behaviour, growth and differentiation. This technique is widely used in developmental biology and neuroscience to investigate how individual cells contribute to the formation and function of tissues and organs.

### P-Element transposons and enhancer traps

P-element transposons are mobile genetic elements that can be used to introduce or disrupt genes within the *Drosophila* genome. These transposons can carry reporter genes, such as GFP, that allow researchers to visualize gene expression patterns or identify enhancer regions that control gene expression [46].

Enhancer traps, which utilize P-element transposons, are used to identify and study regulatory elements in the genome. This technique has been instrumental in mapping the regulatory networks that control development and differentiation in *Drosophila*. Enhancer traps have also been used to study gene expression in disease models, providing insights into how genetic and environmental factors influence disease onset and progression.

## Drosophila genome and its biomedical relevance

**Figure 3.**
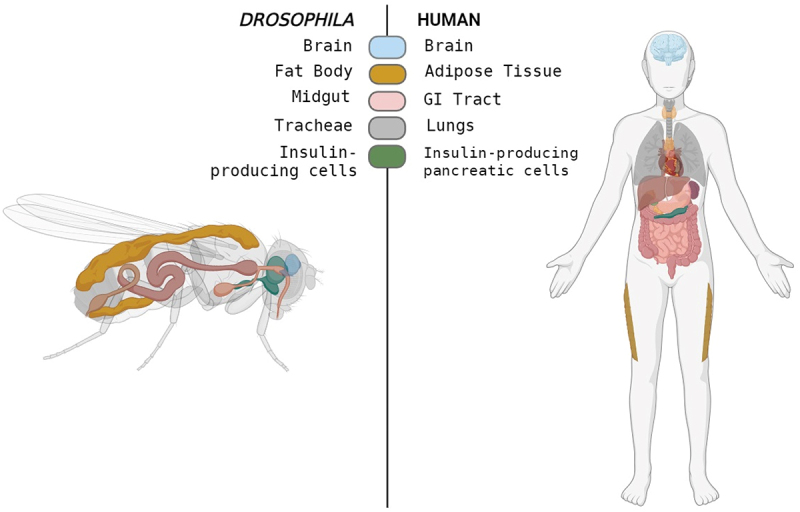
Tissue/Organ functional similarities shared by *Drosophila* and human.

The genome of *Drosophila melanogaster* has been instrumental in genetic and biomedical research, offering valuable insights into fundamental biological processes and disease mechanisms. Comprising approximately 16,000 genes across four pairs of chromosomes, the *Drosophila* genome, despite its relative simplicity compared to the human genome, shares significant genetic homology with humans, with over 60% of its genes having identifiable human counterparts [47,48]. This conservation makes *Drosophila* a powerful model for studying genetically based human diseases. Furthermore, *Drosophila* and humans share notable anatomical similarities in tissues and organs, highlighting the importance of cross-species comparative studies in advancing biological research (Figure 3).

Historically, *Drosophila* has played a pivotal role in the field of genetics. The species gained prominence through the pioneering work of Thomas Hunt Morgan in the early 20th century, who used *Drosophila* to demonstrate the chromosomal theory of inheritance. Morgan’s experiments led to the discovery of X-linked inheritance, establishing the concept that genes reside on chromosomes [49]. This work laid the foundation for modern genetics, and *Drosophila* quickly became a model organism of choice due to its relatively simple genome, short generation time, and ease of genetic manipulation. One of the most significant contributions of *Drosophila* to genetics was the demonstration of x-ray-induced mutations by Hermann Muller in the 1920s. Muller showed that exposure to x-rays increased the mutation rate in *Drosophila*, providing the first evidence that radiation could alter genetic material [50]. This discovery was instrumental in advancing our understanding of mutagenesis and laid the groundwork for radiation genetics, with implications that extended to the study of cancer and other mutation-driven diseases.

The sequencing of the *Drosophila* genome, completed in 2000, marked a major milestone in genomics and provided a comprehensive map of its genetic makeup [47]. This achievement not only underscored the utility of *Drosophila* as a model organism but also facilitated the identification of numerous genes involved in development, behaviour, and disease. The availability of the *Drosophila* genome sequence has enabled researchers to conduct large-scale genetic screens, leading to the discovery of gene functions and interactions that are relevant to human biology. One of the key advantages of the *Drosophila* genome is its relatively small size and high gene density, which simplifies genetic analysis. With only four pairs of chromosomes, *Drosophila* is easier to manipulate genetically than many other model organisms, such as mice. The compactness of the *Drosophila* genome allows for more straightforward mapping of genetic mutations and the identification of gene function through loss-of-function and gain-of-function experiments [51]. Furthermore, the conservation of genetic pathways between *Drosophila* and humans means that findings in *Drosophila* are often directly translatable to human biology.

In the realm of disease modelling, *Drosophila* has been particularly valuable in elucidating the genetic underpinnings of neurodegenerative diseases, cancer, and metabolic disorders. For example, *Drosophila* models of Alzheimer’s disease have been used to study the pathological effects of amyloid-beta and Tau protein accumulation, key features of the disease that are also observed in human patients [52,53]. Similarly, *Drosophila* models have been employed to investigate the role of oncogenes and tumour suppressor genes in cancer, providing insights into the mechanisms of tumorigenesis and metastasis [54]. The simplicity of the *Drosophila* genome, coupled with sophisticated genetic tools such as RNA interference (RNAi) and CRISPR-Cas9, allows for the precise manipulation of genes involved in these diseases, facilitating the development of potential therapeutic strategies [55]. Moreover, the *Drosophila* genome has been a critical resource in studying developmental biology. The discovery of homoeotic genes in *Drosophila*—which control the body plan during embryonic development – has had a profound impact on our understanding of developmental processes. These genes, known as Hox genes, are highly conserved across species and play similar roles in vertebrate development, including humans [56]. The insights gained from studying the *Drosophila* Hox gene cluster have been instrumental in revealing the genetic mechanisms that govern body plan specification and organ development.

The utility of the *Drosophila* genome extends beyond basic research; it also serves as a platform for drug discovery and testing. High-throughput genetic screens in *Drosophila* have been used to identify novel drug targets and to test the efficacy and toxicity of potential therapeutics. For instance, *Drosophila* models of neurodegenerative diseases have been used to screen for compounds that mitigate the toxic effects of protein aggregates, providing leads for the development of drugs for conditions such as Alzheimer’s and Parkinson’s disease [57].

## Applications of Drosophila in disease modeling

The utility of *Drosophila melanogaster* as a model organism extends far beyond basic genetics, providing profound insights into the pathophysiology of various human diseases. Its genetic tractability, combined with a high degree of conservation with human biological pathways, makes *Drosophila* an indispensable tool in modelling diseases such as neurodegenerative disorders, cancer, metabolic diseases and infectious diseases. This section details the specific applications of *Drosophila* in these areas, emphasizing how its unique attributes have advanced our understanding of disease mechanisms and therapeutic approaches.

### Drosophila models of neurodegenerative diseases

Neurodegenerative diseases, characterized by the progressive loss of neuronal structure and function, are among the most studied conditions using *Drosophila* models. *Drosophila* has been instrumental in elucidating the molecular mechanisms underlying diseases such as Alzheimer’s disease, Parkinson’s disease, Huntington’s disease and amyotrophic lateral sclerosis.

#### Drosophila model of Alzheimer’s disease

Alzheimer’s Disease (AD) typically emerges post the age of 65 and represents a variant of dementia. Its hallmark features encompass cognitive decline, alterations in mood and behaviour, accumulation of distinct protein aggregates within the cerebral cortex, alongside volumetric diminishment in brain structures such as the hippocampus and temporal lobes [58–60]. Alzheimer’s Disease stands as a prominent contributor to mortality within the United States. According to statistics derived from the 2010 census, 4.7 million individuals aged 65 and above were afflicted with AD. Projections suggest a substantial escalation, with an estimated 13.8 million Americans anticipated to be affected by AD by the year 2050 [61,62]. While definitive cures for AD remain elusive, available treatment modalities encompass dietary adjustments, lifestyle modifications, and pharmacological interventions aimed at mitigating symptoms and/or slowing disease progression [63,64]. The aetiology of AD remains a focal point of ongoing research, with various hypotheses proposed to elucidate the array of associated risk factors and physiological alterations. Foremost among these is the amyloid hypothesis, positing that the accumulation of distinctive extracellular amyloid – beta (Aβ) aggregates instigate pathology, particularly neurodegeneration. The production of pathogenic, extracellular Aβ42 entails a sequential cleavage process of the intramembrane amyloid precursor protein (APP), referred to as amyloid precursor protein – like (APPL) in *Drosophila*, by two enzymes, BACE1 (β–site APP cleaving enzyme–1) and β–secretase. Conversely, the non-pathogenic cleavage of APP is primarily executed by β–secretase [65–67]. Additionally, another pivotal protein implicated in the amyloid hypothesis is Tau. Under physiological conditions, Tau typically binds to microtubules, contributing to their stability. However, in instances of hyperphosphorylation, Tau undergoes detachment from microtubules, leading to the formation of intracellular aggregates. This process disrupts microtubule stability, consequently impairing neurotransmission. The precise cause of Tau hyperphosphorylation remains elusive; however, emerging evidence suggests potential involvement of amyloid pathology or shared mechanisms such as innate immunity. Certainly, the activation of the innate immune system and the presence of chronic inflammation have been implicated in a range of neurodegenerative disorders [68]. The review by Lye et al. [69] examines the role of *Drosophila* brain immunity concerning both injury and neurodegeneration contexts.

Several alternative hypotheses have been proposed, encompassing various observations associated with AD, including Tau tangles, mitochondrial dysfunction and oxidative stress, inflammation mediated by glial cells, cholinergic dysfunction, toxicity due to metal ions, disturbances in calcium homoeostasis, impaired lymphatic clearance, and vascular dysfunction. These proposed mechanisms are intricately interconnected, frequently through the involvement of Aβ aggregates, and collectively, they contribute to the pathogenesis of AD [66]. Different models of AD in *Drosophila* can be categorized into three main groups: those employing genetic mutations in *Drosophila* genes equivalent to those associated with human diseases, as well as transgenic constructs containing alleles of genes implicated in human disease, and models designed to investigate the impact of environmental stressors on the toxicity of Aβ. (as shown in Table 1). *Drosophila* models have been established for several human genes, including BACE1, CD2AP, ITGAM, XYLT1, BACE2, CELF1, PS2, ITGA9, FERMT2, MEGF10, MAST4, SNRPN, PS1, PTPRD and APP [70,78,80].

**Table 1.** *Drosophila* model of Alzheimer’s disease.

	*DROSOPHILA* MODEL		STAGE OF NEUROPATHOLOGICAL ASSESMENT	ASSAY EMPLOYED FOR NEUROPATHOLOGICAL ASSESSMENT	KEY ACHIEVEMENTS	REFERENCES
***DROSOPHILA* ORTHOLOGS OF HUMAN GENES**						
	Null mutants of APPL		Adult	Histological analysis, phototaxis assay, olfactory acuity assay, shock reactivity, odor conditioning, optomotor assay	Established the role of APPL in brain morphology and behavior, providing insights into neurodegeneration mechanisms	[273]
	Pan-neuronal and photoreceptor-specific expression of *Drosophila* dBACE (*Drosophila* β-secretase) and APPL (Amyloid precursor protein-like) in *Drosophila* results in the production of dAβ (*Drosophila* amyloid beta).		Adult	Histological analysis, Thioflavin S staining, immunohistochemistry, phototaxis assay, TEM	Demonstrated the role of amyloid-beta in retinal degeneration, contributing to understanding AD pathology	[70]
**INCREASED EXPRESSION OF HUMAN TRANSGENES**						
	Expression of Aβ40, Aβ42, and Aβ42arc fused to *Drosophila* Necrotic protein signal peptide (SP) specifically in pan-neuronal cells		Adult	Lifespan measurement, climbing assay, immunostaining, SEM	Identified Aβ42 accumulation’s contribution to AD pathology and its potential as a target for therapeutic intervention	[52]
	Expression of Aβ40 and Aβ42 fused to rat pre-proenkephalin signal peptide (SP) specifically in pan-neuronal and photoreceptor cells.		Larva, Adult	In larvae, immunostaining coupled with confocal microscopy was utilized to visualize Aβ42 accumulation specifically in the imaginal eye discs. For adults, eye morphology was examined using scanning electron microscopy (SEM) and light stereomicroscopy. Lifespan assays were conducted to monitor longevity. Immunostaining with anti-Aβ (6E10) antibodies was employed to detect Aβ42 accumulation in adult eyes. Additionally, toluidine blue histological staining was used to assess the organization of ommatidia in the adult eye tissue.	Demonstrated the role of Aβ42 in eye tissue organization and neurodegeneration, highlighting its potential as a therapeutic target	[53]
	Studying the effects of particular amino acid changes on toxicity by expressing various mutated forms of Aβ42 peptides		Adult	Assessment of lifespan, locomotor function, immunohistochemistry employing anti-Aβ42 antibodies, Thioflavin T staining to quantify rates of Aβ42 aggregation, and transmission electron microscopy (TEM) for examining the morphology of Aβ42 aggregates.	Established the impact of specific mutations on Aβ42 aggregation and neurotoxicity, providing insights into the mechanisms of AD pathology	[71]
	Expression specifically targeted to photoreceptor cells of Aβ42, with an additional blocking function.		Larva, Pupa, Adult	In the third instar larvae stage, immunostaining was conducted to assess eye imaginal disc development and Aβ42 accumulation, while TUNEL staining was utilized to detect cell death in the eye imaginal disc. In the pupal stage, immunostaining was performed to examine eye development and Aβ42 accumulation. Upon reaching adulthood, immunostaining continues to evaluate eye development and Aβ42 accumulation. Additionally, histological analysis was conducted to assess photoreceptor morphology, and SEM was employed to study eye morphology.	Identified the protective effects of targeted blocking functions against Aβ42-induced degeneration in photoreceptor cells, offering potential therapeutic strategies	[72]
	Exploring the effects of specific amino acid substitutions on toxicity through the expression of a variety of mutated Aβ42 peptides		Adult	Lifespan	Clarified the effects of amino acid substitutions on peptide toxicity and aggregation rates, advancing the understanding of mutation-driven neurodegenerative processes	[73]
	Expression of Aβ42 specifically in pan-neuronal and muscle cells, exposure to externally applied Aβ42, and administration of anti-Aβ42 antibody (6E10) treatment		Larva	In third instar larvae, electrophysiology was conducted to assess synaptic transmission, FM1-43 dye imaging was used to visualize neurotransmitter release, and Thioflavin S staining is performed to detect amyloid deposits.	Highlighted the impact of extracellular Aβ42 on synaptic function and the therapeutic potential of anti-Aβ42 antibodies	[74]
	Expression of human amyloid precursor protein (APP) and beta-site amyloid precursor protein cleaving enzyme 1 (BACE1) separately and together specifically in pan-neuronal cells, along with treatment using a γ-secretase inhibitor		Adult	Lifespan assessment, climbing ability, immunostaining, TEM	Revealed interaction between APP and BACE1, informing therapeutic strategies targeting amyloid production	[75]
	Expression of two human Tau variants specifically in pan-neuronal and photoreceptor cells, along with manipulation of light exposure		Adult	Lifespan measurement, histological examination, climbing assay, immunohistochemistry, light microscopy	Provided evidence for Tau-induced neurodegeneration and its modulation by light exposure, guiding future studies on Tau-targeted therapies	[76]
	The presence of human beta-site amyloid precursor protein cleaving enzyme 1 (BACE1) expression and the delayed activation of human amyloid precursor protein (APP) are linked to conditions characterized by late onset		Adult	Measurement of lifespan, climbing ability assessment, immunostaining using anti-Aβ (6E10) to detect amyloid deposition, fluorescence microscopy to identify abnormalities in whole-brain structure	Provided evidence of the role of BACE1 in amyloid deposition and its effects on neurodegeneration and climbing ability	[77]
**INTEGRATION OF *DROSOPHILA* ORTHOLOG MODELS WITH THE OVEREXPRESSION OF HUMAN TRANSGENES**						
	Downregulation of the orthologs corresponding to human SNRPN, FERMT2, ITGA9, CD2AP, CELF1, PTPRD, MAST4, XYLT1, ITGAM in *Drosophila*, while concurrently overexpressing human Tau^V337M^		Adult	Examination of eye morphology using light microscopy	Demonstrated the combined impact of gene downregulation and Tau overexpression on eye morphology, contributing to understanding tauopathies	[78]
	Expression of Aβ42 specifically in pan-neuronal cells, treatment with an iron chelator, and RNA interference (RNAi) targeting ferritin for knockdown		Embryo, Adult	For embryos: conducting a hatching efficiency assay. For adults: performing a survival assay and using Thioflavin T staining to assess amyloid aggregation	Showcased the role of iron in enhancing Aβ42 toxicity and the potential of iron chelation as a therapeutic strategy	[79]
	Overexpression of Aβ42^arc^, inhibition of Draper, and overexpression of Draper/MEGF10		Adult	Assessment of lifespan, Thioflavin S staining and immunostaining using anti-Aβ (6E10) antibody for Aβ detection, climbing assay and histological sectioning for quantifying vacuoles	Highlighted the influence of Draper on Aβ42 toxicity and its regulation of neurodegenerative processes	[80]
	Expression of human Aβ42 specifically in photoreceptor cells within the eyes, along with supplementation with zinc or copper, administration of chelators, and overexpression of MTF-1		Larva, Adult	For larvae: assessment of relative eclosion rate. For adults: examination of ommatidia structure using stereomicroscopy, along with conducting climbing assays.	Identified the role of zinc and copper in Aβ42 toxicity and the neuroprotective effects of metal chelation and MTF-1 overexpression	[81]
	Expression of Aβ42 specifically in photoreceptor cells, modulation of immunophilin expression (both overexpression and underexpression)		Adult	Assessment of lifespan and examination of eye morphology using light microscopy	Demonstrated the impact of immunophilin expression on Aβ42-induced neurodegeneration in photoreceptor cells	[82]

Transgenic constructs have been employed for the purpose of targeting both Aβ production and its toxicity. Additionally, they have been utilized to investigate the involvement of Tau in the pathology of Alzheimer’s disease [52,53,71–74,76,83]. Environmental stressors known to modulate AD progression and beta-amyloid toxicity encompass copper, iron, zinc, and exposure to light [79,81,82,84]. Additionally, *Drosophila* homologs of genes associated with AD have offered valuable insights into both the human genes associated with AD development and the pathways contributing to the disease. In *Drosophila* models of AD, the gene Draper, equivalent to MEGF10 in humans, is involved in the glial engulfment of amyloid-beta (Aβ), consequently reducing neurotoxicity [80]. In a separate study examining 87 *Drosophila* genes, each possessing a human homolog identified in Genome-Wide Association Studies (GWAS) as an AD-associated genomic locus, nine genes were found to notably influence Tau toxicity. These genes include SNRPN (SmB), FERMT2 (Fit 1, Fit 2), ITGA9 (scb), CD2AP (cindr), MAST4 (CG6498), XYLT1 (oxt), ITGAM (scb), CELF1 (aret), and PTPRD (Lar) [78]. The proteins encoded by FERMT2 and CD2AP both participate in cell adhesion and signalling processes alongside integrins. Furthermore, ITGA9 and ITGAM are responsible for producing α-subunits essential for integrin receptor function. Additionally, XYLT1 and PTPRD are involved in cell adhesion mechanisms as well [78,85–88].

The human peptide Aβ42 is renowned for its propensity to aggregate and form extracellular plaques in AD. Transgenic *Drosophila* models have incorporated human Aβ42 fused with diverse signal peptides to aid in secretion. These models have been targeted using an anti-Aβ42 antibody and have been engineered to express single amino acid substitutions anticipated through computer modelling [52,53,71,73,74].

Additionally, within human physiology, the protein produced by the APP gene is responsible for transporting the Aβ peptide and undergoes cleavage by both BACE1 and β-secretase prior to its release into the extracellular space. Transgenic arrangements in *Drosophila* have been utilized to investigate the functions of BACE1, APP, and pathogenic Psn (the *Drosophila* counterpart of a β-secretase element) both separately and in concert [75,89–91]. Environmental influences, such as dietary habits, lifestyle choices, and exposure to various chemicals, have been identified as significant contributors to Alzheimer’s disease in human populations [92,93]. Research utilizing *Drosophila* models of Alzheimer’s disease has investigated the impact of dietary metals like iron, copper, and zinc through manipulation of exposure levels using diverse methodologies [94,95]. Supplementing copper and zinc has been shown to worsen the toxicity of Aβ42, whereas employing chelators, enhancing the expression of detoxifying proteins, and upregulating the expression of transport proteins have been demonstrated to mitigate this toxicity [81,82]. Overexpression of iron chelators has been found to mitigate Aβ42 toxicity, whereas reducing the expression of these chelators leads to an increase in toxicity [79,84]. Examining a distinct facet of lifestyle and environmental influence, a notable study utilizing a Tau model of Alzheimer’s disease discovered that perturbation of the circadian rhythm through exposure to dim light resulted in heightened neurodegeneration [76].

#### Drosophila model of Parkinson’s disease and Lewy Body Dementia

Lewy Body Dementias (LBDs) are neurological disorders distinguished by the presence of α-synuclein (α-syn) aggregates within brain cells. The accumulation of α-synuclein aggregates, known as Lewy bodies, characterizes Lewy Body Dementias (LBDs). This umbrella term encompasses two primary types: Parkinson’s disease (PD) and Dementia with Lewy Bodies (DLB). While overexpression of α-synuclein (α-syn) in *Drosophila* can potentially model both Parkinson’s disease and Dementia with Lewy Bodies (DLB), the existing literature primarily categorizes such models as PD models. However, it’s essential to note that these models may also provide insights into DLB pathogenesis due to the shared underlying pathology of α-syn accumulation in both PD and DLB. Parkinson’s disease is indeed a neurodegenerative disorder primarily affecting individuals over the age of 45. In North America, the incidence rate of PD is estimated to be approximately 572 cases per 100,000 individuals in this age group. The projected number of individuals diagnosed with Parkinson’s disease in the United States was anticipated to reach 930,000 by the year 2020 [96]. The hallmark symptoms of Parkinson’s disease encompass tremor and postural instability, which arise from the degeneration of midbrain dopaminergic (DA) neurons responsible for supplying dopamine to the basal ganglia [97]. In addition to the basal ganglia, Parkinson’s disease also impacts other brain structures including the cerebral cortex, olfactory tubercle, as well as post-commissural putamen, giving rise to a range of diverse symptoms [97]. While there are currently no known cures for Parkinson’s disease (PD), medications that target dopamine receptors, such as dopamine and levodopa, have demonstrated efficacy in alleviating symptoms [98]. Additionally, non-pharmacological treatments like deep brain stimulation and exercise therapy have shown promise in managing PD symptoms [99].

Among the molecular mechanisms implicated in Parkinson’s disease (PD) pathology are oxidative stress, neuroinflammation, disturbances in calcium homoeostasis, disruptions in α-synuclein proteostasis, defects in axonal transport, and mitochondrial dysfunction [98]. Due to the multifactorial nature of Parkinson’s disease aetiology, researchers have developed utilizing various *Drosophila* models to replicate established contributing factors (as shown in Table 2). Investigations in *Drosophila* models of Parkinson’s disease have explored orthologous genes, constructs bioengineered to carry human genes, as well as environmental factors, aiming to elucidate the complex aetiology of the disease.

**Table 2.** *Drosophila* model of Parkinson’s disease.

	*DROSOPHILA* MODEL		STAGE OF NEUROPATHOLOGICAL ASSESMENT	ASSAY EMPLOYED FOR NEUROPATHOLOGICAL ASSESSMENT	KEY ACHIEVEMENTS	REFERENCES
***DROSOPHILA* ORTHOLOGS OF HUMAN GENES**	Mutants with alterations in the PINK1 gene and reduction of PINK1 expression specifically in dopamine neurons		Adult	Measurement of lifespan, immunostaining for tyrosine hydroxylase (TH), chemotaxis assay, dopamine enzyme immunoassay, high-performance liquid chromatography (HPLC) for dopamine tissue and dopamine levels	Showed the link between PINK1 mutations and dopamine neuron degeneration, mimicking Parkinson’s disease pathology	[100], [101]
	Mutants with alterations in the parkin gene		Adult	Immunostaining for TH and conducting a climbing assay	Established the effects of parkin gene alterations on dopaminergic neuron health and motor function, aiding in Parkinson’s disease studies	[102]
	LRRK2 mutants		Adult	Assessment of climbing ability and immunostaining for TH	Explored the impact of LRRK2 mutations on dopaminergic neuron function and motor deficits relevant to Parkinson’s disease	[103]
	Reduction of HtrA2 expression specifically in dopamine neurons and photoreceptor cells		Adult	Assessment of lifespan, climbing ability, and scanning electron microscopy (SEM) for eye morphology	Highlighted HtrA2’s role in maintaining dopaminergic and photoreceptor cell health, contributing to insights on neurodegenerative diseases	[104]
	Mutations in both CG31414 and CG31148 genes, known as double heterozygous GBA mutants		Adult	Measurement of lifespan, immunostaining for TH, and climbing assay.	Demonstrated the effects of GBA mutations on lifespan and dopaminergic function, linking to Parkinson’s disease	[105]
	Mutations in the DJ-1 gene subjected to exposure to rotenone, hydrogen peroxide, and paraquat		Adult	Measurement of lifespan and immunostaining for TH	Established the role of DJ-1 in oxidative stress responses and dopaminergic neuron survival, aiding in Parkinson’s disease research	[106]
	Overexpression of dUCH specifically in photoreceptor cells and knockdown of dUCH specifically in dopamine neurons		Larva, Pupa, Adult	SEM for examining eye morphology, immunostaining for activated-Caspase 3 and TH	Examined the effects of dUCH expression alterations on neurodegeneration in dopaminergic and photoreceptor cells	[107]
**OVEREXPRESSION OF HUMAN TRANSGENES**	Simultaneous expression of Tau and Alpha-Synuclein (α-syn)		Larva, Adult	Immunostaining for activated-caspase 3, NMJ morphology, immunostaining for TH, SEM for adult eye morphology	Showed the synergistic effects of Tau and α-syn on neurodegeneration, providing a model for studying combined pathologies	[108]
	Expression of LRRK2 and LRRK2-G2019S-2 in pan-neuronal cells, photoreceptor cells and dopamine neurons		Adult	Lifespan measurement, climbing assay, photoreceptor morphology, TEM, immunostaining for TH, actometer test	Identified the role of LRRK2 mutations in dopaminergic neurodegeneration, aiding in understanding Parkinson’s disease	[109]
	Increased expression of Pael-R specifically in dopamine neurons		Adult	Immunostaining for TH	Established the link between Pael-R overexpression and dopaminergic neuron degeneration, providing a model for Parkinson’s disease	[109]
**EXPOSURE TO TOXINS**	Rotenone		Adult	Immunostaining for TH, climbing assay	Demonstrated the role of environmental toxins like Rotenone in Parkinson’s disease pathogenesis	[110]
	Paraquat		Adult	Immunostaining for TH, climbing assay, lifespan, jumping assay, dopamine levels	Confirmed the role of oxidative stress in Parkinson’s disease, showing how Paraquat induces dopaminergic neuron degeneration	[111]; [112]

The genes associated with Parkinson’s disease and possessing utilizable homologs in *Drosophila* comprise PARK2, DJ-1, HtrA2, Tau, PINK1, GBA, UCH-L1, and LRRK2 [100,104,105,113]. Alpha-Synuclein (α-syn) and Pael-R lack homologs in *Drosophila* and are investigated through transgenic models [39,109,114]. Additionally, human transgenes for genes such as LRRK2 and Tau have been introduced into *Drosophila* models [108,109,115]. Furthermore, environmental stressors, including commonly used pesticides like paraquat and rotenone, have been evaluated using *Drosophila* models [110–112].

The functions of *Drosophila* orthologs of genes linked to Parkinson’s disease (PD) can be explored through various methods such as utilizing mutant flies or employing tissue and/or cell-specific overexpression or knockdown approaches facilitated by binary expression systems [38]. The PARK2 gene encodes the Parkin protein, responsible for tagging abnormal proteins for degradation. PD-related proteins like Pael-R and α-synuclein are among those monitored by PARK2 [109]. Notably, *Drosophila* brains, akin to human brains, contain dopaminergic (DA) neurons. Hence, it is possible to investigate the ramifications of specific mutations and gene overexpression in dopaminergic (DA) neurons. In *Drosophila*, the PINK1 protein plays a crucial role in mitochondrial function. Mutants of PINK1 display a reduction in dopaminergic (DA) neurons and demonstrate impairments in both olfactory function and motor abilities [100,101,114,116]. Loss-of-function mutations in *Drosophila* LRRK2 similarly decrease the number of dopaminergic (DA) neurons and result in impaired locomotor activity [103]. Specifically, knockdown of the *Drosophila* ortholog of dUCH, UCH-L1, restricted to DA neurons leads to a Parkinson’s disease-like phenotype characterized by the loss of DA neurons. Conversely, overexpression of dUCH results in abnormal patterning of the pupal retina, caspase-dependent cell death in eye imaginal discs, also a rough eye phenotype in adults was observed [107].

The HtrA2 protein, possessing protease activity and participating in apoptosis, when its function is knocked down in *Drosophila* dopaminergic (DA) neurons and photoreceptor cells, results in reduced lifespan, impaired motor function, and a decrease in the number of ommatidia [104]. The GBA gene encodes the enzyme glucocerebrosidase, which plays a crucial role in preventing the accumulation of glucosylceramides. Mutations in the GBA gene in *Drosophila* lead to dopaminergic (DA) cell death, motor impairments, and reduced lifespan [105]. Additionally, the Tau protein is capable of forming neurotoxic inclusions implicated in both Parkinson’s disease (PD) and Alzheimer’s disease (AD) [113].

Overexpression of *Drosophila* Tau specifically in mushroom body neurons leads to learning and memory deficits [117]. In *Drosophila*, both overexpression and underexpression of LRRK2 exacerbate Tau toxicity, leading to the loss of tyrosine hydroxylase (TH)-immunoreactive neurons [118]. Since *Drosophila* lacks orthologs of human α-synuclein or Pael-R, investigations into their functions in *Drosophila* have utilized the overexpression of human cDNAs. Overexpression of various human α-synuclein variants in *Drosophila* results in locomotor abnormalities, formation of Lewy bodies in the brain, and degeneration of the retina [39]. In a *Drosophila* model, simultaneous expression of the human proteins α-synuclein and Tau leads to the formation of inclusions containing ubiquitinylated proteins, which interfere with cytoskeletal functions and ultimately result in neurodegeneration [108]. Overexpression of both wild-type and mutant forms of human LRRK2 in *Drosophila* results in degeneration of photoreceptor cells and neurons, accompanied by symptoms such as motor deficits and reduced lifespan [115]. Rotenone and paraquat, pesticides associated with Parkinson’s disease (PD) development in humans, have been studied using *Drosophila* models of PD to elucidate the underlying mechanisms [110,111,112,119]. Rotenone functions by inhibiting mitochondrial Complex I, which subsequently induces oxidative stress. In *Drosophila*, exposure to rotenone leads to dose-dependent symptoms such as motor impairments and the selective loss of dopaminergic (DA) neurons. Additionally, it has been observed that while the Parkinson’s disease medication L-dopa can effectively alleviate motor deficits, it does not prevent the loss of DA neurons [110]. Exposure to paraquat in *Drosophila* also induces oxidative stress and leads to the loss of dopaminergic (DA) neurons [112]. These alterations mirror the findings observed in post-mortem samples from Parkinson’s disease patients exposed to paraquat [120]. Recent studies conducted in *Drosophila* have revealed that exposure to paraquat not only induces oxidative stress and dopaminergic (DA) neuron loss but also results in deregulated innate immune responses [119]. While it remains uncertain whether deregulation of the innate immune response serves as a primary driver of neurodegeneration following paraquat exposure, it’s worth noting that activation of the innate response has been associated with neurodegeneration in other scenarios (e.g. [121]).

#### Drosophila model of Huntington’s disease

Huntington’s disease (HD), similar to some other neurodegenerative disorders, results from a repeat expansion mutation, specifically a CAG trinucleotide repeat that encodes a polyglutamine (polyQ) sequence of 36 or more units within the Huntingtin (HTT) protein [122,123]. Typically diagnosed between the ages of 30 and 50, Huntington’s disease manifests with progressive challenges in coordination, cognitive function, decision-making, and mood regulation. However, its onset can occur earlier with varying manifestations [124]. Prevalence rates of Huntington’s disease vary among different populations, with higher occurrences observed in Australian, European, as well as the North American populations at 5.7 cases per 100,000 individuals, compared to 0.4 cases per 100,000 individuals in Asian populations [125]. Following diagnosis, individuals with HD typically have a life expectancy of only 17–20 years. Presently, no treatments are known to halt the progression of the disease; however, certain therapies can manage symptoms such as chorea [124,125]. Since the *Drosophila* Huntingtin (dHtt) lacks expanded polyglutamine (polyQ) segments in its amino terminus [126], many *Drosophila* models of Huntington’s disease (HD) involve the transgenic introduction of the mutant human gene, primarily concentrating on extensive polyQ domains rather than examining the entirety of the protein (Table 3). The divergence in *Drosophila* models of HD largely hinges on the specific segments of the Huntingtin (Htt) protein that are expressed. In certain investigations, researchers have utilized fragments of the gene, such as solely expressing exon one or the initial three exons. Conversely, other studies have employed larger segments, such as a 12-exon fragment or the sequence encompassing the entire protein [128,134–136]. To assess compounds of interest in HD models, various approaches have been employed. These include transgenic expression of polyglutamine aggregation inhibitors like QBP1 (polyglutamine binding peptide) and bivalent polyQ peptides, overexpression of genes such as NMNAT (nicotinamide mononucleotide adenylyltransferase), administration of HDAC (histone deacetylase) inhibitors via dietary intake, and delivery of polyQ aggregation inhibitors using nanoparticles [128,131,132,137]. Certainly, in addition to overexpressing genes like NMNAT, which facilitates the reduction of mutant Huntingtin (Htt) aggregation by stimulating autophagic clearance, the utilization of loss-of-function mutations and conditional expression (initiated after symptom onset) has demonstrated utility in examining pathology and potential treatment strategies [132]. Research has demonstrated that treatment with HDAC inhibitors can effectively halt polyglutamine-induced toxicity and ameliorate lethality. Furthermore, assays such as survival assessments, photoreceptor quantification, circadian rhythmicity evaluation, and motor performance tests serve as effective screening methods for treatments or deficiency mutations [135,136]. Although not a direct method for the assessment of neuropathology, alterations in circadian rhythms are highly correlated with neurodegeneration in both humans and animal models [138,139].

**Table 3.** *Drosophila* model of Huntington’s disease.

		*DROSOPHILA* MODEL	STAGE OF NEUROPATHOLOGICAL ASSESMENT	ASSAY EMPLOYED FOR NEUROPATHOLOGICAL ASSESSMENT	KEY ACHIEVEMENTS	REFERENCES
***DROSOPHILA* ORTHOLOGS OF HUMAN GENES**		*Drosophila* huntingtin protein (dHtt) lacks a polyglutamine (polyQ) tract in its N-terminus	N/A	N/A	Highlighted the lack of polyQ in dHtt, providing a baseline for comparison with toxic polyQ models in Huntington’s disease research	[126]
**OVEREXPRESSION OF HUMAN TRANSGENES**		Q48 constructs transngenic expression	Adult	Assessment of locomotor activity and counting of photoreceptor morphology	Showed the impact of Q48 expression on locomotor and photoreceptor morphology, relevant to Huntington’s disease	[127]
		Transgenic expression of either a Q48 peptide or the Htt Exon1p specifically in neurons	Adult	Measurement of lifespan and counting of photoreceptor morphology	Investigated the neurotoxic effects of Q48 and Htt Exon1p on neuronal and photoreceptor health	[128]
		Q48 and Q108 transgenic expression peptides, as well as bivalent polyQ peptides transgenic expression	Adult	Measurement of lifespan and counting of photoreceptor morphology	Explored the varying impacts of different polyQ lengths on lifespan and photoreceptor integrity	[129]
		Production of Q20 and Q127 peptides	Adult	Scanning electron microscopy (SEM) and light microscopy for examining retina morphology, light microscopy for detecting pigmentation defects, and staining with FITC to identify the presence of polyQ aggregates	Demonstrated the differential impacts of Q20 and Q127 on retinal morphology and polyQ aggregate formation, highlighting the significance of polyQ length in disease pathology	[130]
**OVEREXPRESSION OF HUMAN TRANSGENES**		Expression of peptides containing 93 glutamine repeats (Q93) and 20 glutamine repeats (Q20)	Larva, Adult	In adults: Assessment of locomotion In larvae: Conducting a crawling assay	Showed the effects of varying polyQ lengths on locomotor abilities, providing insights into polyglutamine toxicity in Huntington’s disease	[131]
		Expression of mRFP-tagged N-terminal fragments derived from human peptides containing either 15 glutamine repeats 138 glutamine repeats (Q138) or (Q15).	Adult	Measurement of lifespan, assessment of locomotion, immunostaining for activated-Caspase 3 to detect apoptosis, and immunostaining to evaluate brain size.	Highlighted the role of extended polyQ repeats in neurodegeneration and brain size reduction, offering valuable insights into Huntington’s disease mechanisms	[132]
		Expression of an mRFP-tagged N-terminal fragment derived from human peptides containing either 15 glutamine repeats (Q15) or 138 glutamine repeats (Q138), encompassing exons 1-12	Adult	Immunofluorescence was conducted to observe the spread of Huntingtin aggregates in the brain	Provided direct visualization of Huntingtin aggregate formation, enhancing the understanding of aggregate dynamics in Huntington’s disease	[133]
		Expression of full-length human Htt containing either 128 glutamine repeats (Q128) or 16 glutamine repeats (Q16)	Larva, Adult	In adults: Conducting a Western blot to assess Huntingtin levels, counting photoreceptor morphology, evaluating locomotion and flying ability, using confocal microscopy to count neuronal projections into indirect flight muscles (IFMs). In larvae: Performing immunohistochemistry to count third-instar larval neuromuscular junctions (NMJs), measuring excitatory junction potential (EJP) amplitudes, and conducting Ca^2+^ imaging.	Highlighted the differential effects of polyQ lengths on neuronal function and neuromuscular junction integrity, linking these effects to Huntington’s pathology	[134]
		Temperature-inducible expression of either a 12-exon fragment of the human Htt gene containing Q15 or Q138 repeats, or expression of a 548 amino acid segment of human Htt with either no glutamine repeats (Q0) or 128 glutamine repeats (Q128)	Larva, pharate adult, and Adult	In adults: Utilization of an RFP tag for imaging of Htt aggregation and localization. In pharate adults: Examination of lethality. In larvae: Assessment of viability beyond the 2nd instar for a small molecule screen, and employing Fluorescence Recovery After Photobleaching (FRAP) for monitoring aggregate growth	Provided insights into Htt aggregation dynamics and the impact of polyQ expansion on lethality and viability in Huntington’s models	[135]

#### Drosophila model of frontotemporal dementia and amyotrophic lateral sclerosis

Amyotrophic lateral sclerosis (ALS), often known as Lou Gehrig’s disease, is defined by the progressive breakdown of motor neurons. ALS is considered a relatively rare but rapidly progressing neurodegenerative disease, typically resulting in death within approximately 2 to 5 years from the time of diagnosis. Familial ALS (FALS) constitutes approximately 10% of all ALS cases, with the remainder of 90% being represented by sporadic ALS (SALS) [140]. Several genes have been associated with ALS, and seven of these genes have been utilized to develop *Drosophila* models of ALS (as shown in Table 4). They include: TDP-43, UBQLN2, C9ORF72, SOD-1, VAPB, VCP, and FUS. Productive utilization of *Drosophila* models of ALS has involved various approaches, including reduced expression, overexpression, as well as expression of mutant versions of genes associated with the disease. Various assessment methods have been utilized, encompassing the measurement of lifespan, evaluation of locomotor activity and analysis of neuromuscular junction (NMJ) phenotypes. Frontotemporal dementia (FTD) comprises a spectrum of disorders distinguished by the deterioration of the frontal and temporal lobes of the brain. It frequently manifests with an early onset. Genes implicated in contributing to frontotemporal dementia (FTD) include FUS, MAPT/tau, PGRN, C9ORF72, TDP-43, TMEM106B, VCP, and CHMP2B (as reviewed in [160]. Notably, there is overlap with other neurodegenerative diseases such as ALS (TDP-43, C9ORF72, VCP, and FUS), Alzheimer’s disease (Tau), and Parkinson’s disease (Tau). The primary contributing factor to ALS is a particular repetitive expansion found within the C9ORF72 gene, which consists of hundreds or thousands of intronic hexanucleotide repeats, denoted as (G4C2)n [161,162]. Hexanucleotide repeat expansion (HRE) has been detected in over 5% of sporadic ALS (SALS) patients and 39% of white American and European familial ALS (FALS) patients, although its prevalence may vary in other ethnic populations [163]. Repeat RNA sequences have been identified as neurotoxic agents. However, repeat-associated non-AUG (RAN) translation from these RNA sequences can also generate dipeptide repeat (DPR) proteins, which possess neurotoxic properties [164,165].

**Table 4.** *Drosophila* models of frontotemporal dementia and amyotrophic lateral sclerosis.

	*DROSOPHILA* MODEL		STAGE OF NEUROPATHOLOGICAL ASSESMENT	ASSAY EMPLOYED FOR NEUROPATHOLOGICAL ASSESSMENT	KEY ACHIEVEMENTS	REFERENCES
***DROSOPHILA* COUNTERPART OF THE HUMAN GENE FUS**	Presentation of wildtype and mutated FUS gene variants		Larva, Adult	Immunostaining to identify changes in the subcellular distribution of Cabeza in larval motor neurons, assessment of adult eye morphology, and measurement of lifespan.	Showed the impact of FUS mutations on neuronal health and lifespan, highlighting FUS’s role in neurodegenerative processes	[141]
**INCREASED EXPRESSION OF HUMAN TRANSGENES, SPECIFICALLY C9ORF72**	Expression of UAS-(G4C2)3 and UAS-(G4C2)30 constructs in both eye and motor neurons, specifically targeting pan-neuronal cells		Adult	Assessment of lifespan, examination of eye structure and ommatidia loss using light and SEM, and locomotion assay	Demonstrated the contribution of G4C2 repeats to neuronal degeneration and locomotor dysfunction, relevant to C9orf72-linked diseases	[142]
	Expression of RNA-only constructs containing (G4C2)106 repeats, encompassing both intronic (nucleus) and polyadenylated (cytoplasmic) sense and antisense transcripts, specifically in pan-neuronal cells. Additionally, pan-neuronal expression of UAS-RNA sense polyA constructs containing 800-1000 and greater than 1000 (G4C2) repeats		Adult	Measurement of lifespan, assessment of negative geotaxis (climbing ability), and examination of eye morphology using light microscopy.	Highlighted the role of G4C2 RNA repeats in neurodegeneration, emphasizing RNA toxicity mechanisms in ALS/FTD pathology	[143]
	Expression of UAS constructs containing 3, 36, and 103 pure repeats, and 36, 108, and approximately 288 RNA-only (G4C2) repeats in both eye and pan-neuronal cells		Embryo, Adult	Examination of eye structure using stereomicroscopy, assessment of lifespan, and determination of egg-to-adult viability	Showed the effects of varying repeat lengths on eye structure and survival, linking these effects to repeat expansion disorders	[144]
	Expression of UAS-(G4C2)48 specifically in Class IV epidermal sensory dendritic arborization neurons		Larva	Analysis of dendritic branching using confocal microscopy	Identified dendritic abnormalities associated with G4C2 repeats, providing insights into C9orf72-linked neurodegeneration	[145]
	Abnormal expression of UAS constructs containing 30 (G4C2) repeats		Larva, Adult	Assessment of nuclear import and examination of adult eye morphology.	Demonstrated the effects of G4C2 repeats on nuclear import and eye morphology, suggesting links to cellular stress and degeneration	Zhang et al., 2015
	Abnormal expression of UAS constructs containing 8, 28, and 58 (G4C2) repeats		Larva, Adult	Assessment of larval locomotion, examination of larval salivary gland nuclear envelope morphology, and evaluation of adult eye morphology.	Highlighted the impact of G4C2 repeats on locomotion and nuclear morphology, advancing the understanding of repeat expansion toxicity	[146]; [147]
**VCP**	Expression of wild-type and mutated VAP-33		Larva, *Drosophila* cell culture and Adult	For larvae: Examination of larval wing imaginal discs, larval neuromuscular junctions. For adults: Analysis of eye morphology, assessment of cell death, and investigation of ubiquitinated aggregates. For *Drosophila* cell culture: Assessment of ER stress in *Drosophila* S2 cell culture.	Demonstrated the effects of VAP-33 mutations on cellular stress responses and neuronal health, contributing to ALS research	[149]; [150];[148]
**TDP–43**	Abnormal expression of both wild-type and disease-mutated variants		Cultured motorneurons, Larva, and Adult	Examination of larval neuromuscular junction (NMJ) morphology, assessment of larval motor neuron death, investigation of larval glia, and analysis of adult sleep patterns.	Showed the impact of TDP-43 mutations on neuronal and glial cells, linking molecular changes to behavioral deficits	[151]
	Decreased and abnormal expression of wild-type TDP-43		Larva, Adult	Assessment of larval and adult locomotion, examination of larval NMJ morphology, evaluation of adult mushroom body morphology, and testing of adult learning abilities.	Highlighted the role of TDP-43 in neural function and neurodegeneration, with specific impacts on cognition and mobility	[153]
	Abnormal expression of both wild-type and disease-mutated variants		Larval eye imaginal discs, Adult	Investigation of subcellular localization, assessment of lifespan, and measurement of locomotor activity.	Showed the impact of TDP-43 mutations on neuronal and glial cells, linking molecular changes to behavioral deficits	[152]
**INCREASED EXPRESSION OF HUMAN TRANSGENES, SPECIFICALLY UBQLN1/2**	Simultaneous expression of human TDP-43 and UBQLN		Larva, Adult	Assessment of NMJ morphology, measurement of lifespan, quantification of TDP-43 levels in lysates obtained from adult head, examination of adult eye morphology, and conducting locomotion assays in adults.	Showed how UBQLN modulates TDP-43 toxicity, providing potential targets for therapeutic intervention in TDP-43-associated disorders	[76]
	Abnormal expression of both wild-type and disease variants		Adult	Quantification of TDP-43 levels in lysates obtained from adult eyes	Highlighted TDP-43 accumulation in neurodegenerative pathology, with implications for disease progression in ALS/FTD models	[154]
**FUS**	Expression of both wildtype and disease-mutated FUS specifically in motor neurons		Larva, Adult	Assessment of larval brain size, subcellular localization of motor neurons in larvae, evaluation of larval locomotion, and examination of adult eye morphology.	Identified the impact of FUS on neuronal health and motor function, advancing knowledge of FUS-related neurodegeneration	[155]
	Abnormal expression of both wildtype and disease-mutated FUS		Adult	Evaluation of adult eye morphology	Demonstrated the effects of FUS mutations on eye morphology, offering insights into the impact of FUS in neurodegenerative diseases	[157]; [156]
**SOD–1**	Abnormal expression of both wild-type and disease variants		Adult	Measurement of lifespan, assessment of locomotion, quantification of motor neurons, evaluation of neuronal accumulation of SOD-1, and examination of glial stress response	Demonstrated the impact of abnormal SOD-1 expression on motor neuron function and glial response, linking these changes to ALS pathology	[158]
**VAPB**	Expression of wild-type human VAPB specifically in *Drosophila* neurons		Larva	In larvae: Restoration of viability, examination of NMJ morphology, and assessment of NMJ electrophysiology in loss-of-function mutations of *Drosophila* VAP-33	Revealed the critical role of VAPB in NMJ function and viability, emphasizing its importance in neuromuscular disorders	[159]

In *Drosophila*, various strategies have been employed to introduce precise G4C2 repeats and explore potential mechanisms of neurotoxicity (Table 4). In a particular study, it was demonstrated that even as few as 30 repeats of the G4C2 sequence were adequate to induce neurodegeneration [142]. In a subsequent investigation, various RNA-only expression methods were compared, achieved by inserting stop codons to hinder dipeptide repeat (DPR) protein synthesis. Remarkably, the RNA containing the hexanucleotide repeat expansion (HRE) did not exhibit toxicity in this study, leading to the conclusion that the DPR proteins encoded by the hexanucleotide repeats likely mediate neurotoxicity [143]. In line with this observation, a comparison of the effects of expressing RNA encoding various dipeptide combinations without utilizing the G4C2 motif revealed that only dipeptide repeat (DPR) proteins containing arginine were neurotoxic [144]. The findings from *Drosophila* studies stand in contrast to results observed in zebrafish, where both dipeptide repeat (DPR) proteins and clusters of the mutant RNA were found to be neurotoxic [166,167]. Post-mortem examinations of ALS patients commonly reveal the presence of both protein and RNA aggregates in motor neurons. Moreover, these aggregates frequently exhibit the presence of both ubiquitin and TDP-43, thus linking multiple ALS-associated genes in a shared, proteostasis-defective programme. TDP-43 is responsible for encoding the transactive response (TAR) DNA-binding protein, which has the ability to bind to both DNA and RNA. Mutations in TDP-43 contribute to approximately 4% of familial ALS (FALS) cases. The TDP-43 protein is typically localized to the nucleus under normal conditions. However, in approximately 90% of ALS patient samples, TDP-43 is found to localize to the cytoplasm instead. Certainly, cytoplasmic aggregates of TDP-43 are detected in approximately 90% of sporadic ALS (SALS) brain and spinal cord specimens, rendering these aggregates one of the most dependable diagnostic markers for ALS [168]. TDP-43 is classified as a heterogeneous nuclear ribonucleoprotein (hnRNP) and is known to play roles in various cellular processes including transcription, mRNA splicing, as well as the transport of mRNA. The *Drosophila* ALS models offer distinct and potent tools for unravelling the underlying causes of ALS. Advanced genetic analyses, which are often impractical in other model systems, have enabled the identification of both non-autonomous and cell-intrinsic pathways leading to neurotoxicity [169]. Moreover, these analyses have facilitated the differentiation between the contributions of proteins and RNA to neurotoxicity [143,144]. Furthermore, the utilization of advanced genetic methodologies has facilitated the discovery of interacting genetic regions associated with established ALS genes [170–174]. The genetic interplays identified have yielded valuable insights into the molecular pathways associated with neurodegeneration in individuals affected by ALS, thus furnishing a foundation for evaluating prospective ALS treatment options [175].

#### Drosophila model of traumatic brain injury

In 2013, the Wassarman and Ganetzky laboratories introduced the initial *Drosophila* model of closed-head traumatic brain injury (TBI), as outlined in Katzenberger et al.‘s publication [176] (Table 5). Like in humans, TBI in *Drosophila* results in temporary incapacitation, ataxia, activation of the innate immune response, neurodegeneration, and eventual mortality [176]. The neurodegeneration observed in *Drosophila* TBI models is akin to chronic traumatic encephalopathy (CTE) seen in human TBI patients. Over the past seven years, significant progress has been made in understanding the factors that influence TBI outcomes in *Drosophila*. These factors include age, diet, and genetic background, as elucidated in studies by Katzenberger et al. in 2015 and 2016. The ability to investigate the mechanisms driving neurodegeneration within controlled genetic backgrounds is immensely powerful and is already yielding understanding of both genetic and environmental f factors that may contribute to neurodegeneration or confer neuroprotection. The standard TBI protocol in *Drosophila* typically entails administering four impacts spaced at 5-minute intervals. A common outcome measure is the percentage of injured flies that perish within the initial 24 hours following the injury. A survey conducted on over 200 ‘wild type’ *Drosophila* strains, originating from a single wild type population [178], unveiled that post-TBI mortality is significantly influenced by genetic background. Some strains displayed as low as 10% mortality, while others showed up to 60% mortality [179]. Furthermore, utilizing mortality as a metric, TBI outcomes were observed to be more adverse in older adults compared to younger adults [180]. Remarkably, limiting food intake following TBI was demonstrated to yield beneficial effects, mirroring TBI outcomes in humans. Increased hyperglycaemia, as observed in patients with diabetes, is notably associated with a substantially heightened risk of mortality after TBI [179]. These findings imply that the secondary injuries culminating in organismal demise exhibit parallels between *Drosophila* and humans. Therefore, further investigations in *Drosophila* are poised to furnish additional novel insights, aiding in the comprehension of the intricate repercussions of traumatic brain injury [179]. Gene expression analyses have facilitated the identification of genes that are either upregulated or downregulated following *Drosophila* TBI. The upregulated genes encompass components of the *Drosophila* innate immune system, as delineated by Katzenberger et al. in [180]. Interestingly, some of these genes have previously been associated with neurodegeneration in *Drosophila*, as evidenced by studies conducted by Cao et al. in 2013 and Kounatidis et al. in [181]. This observation raises the intriguing potential for pharmaceutical intervention targeting innate immunity pathways in human patient might attenuate secondary injuries, potentially averting adverse outcomes associated TBI. In recent years, the effectiveness of this model has become evident to the extent that other research laboratories have begun to adopt it [182–186]. Due to the similarities between *Drosophila* and human responses to TBI, this model holds promise for various future applications. These applications encompass assessing the efficacy of diverse drugs in treating TBI in clinical settings, as indicated by research conducted by Sanuki et al. in [177]. Future applications of this research will involve evaluating the effectiveness of these drug compounds in averting genetically triggered neurodegeneration. Furthermore, given that TBI patients often necessitate surgery, not only for the head injury but also for concurrent injuries, the *Drosophila* model is anticipated to be valuable for assessing the safety of specific anaesthetics for TBI patients, as highlighted in research by Fischer et al. in [187] (Table 5).

**Table 5.** *Drosophila* model of traumatic brain injury.

*DROSOPHILA* MODEL	STAGE OF NEUROPATHOLOGICAL ASSESMENT	ASSAY EMPLOYED FOR NEUROPATHOLOGICAL ASSESSMENT	KEY ACHIEVEMENTS	REFERENCES
Injury caused by the High-Impact Trauma device	Adult	Assessment of lifespan and histological staining to quantify vacuoles	Demonstrated the effects of traumatic brain injury on lifespan and brain morphology, providing a model for studying TBI outcomes	[176]
Stab injury inflicted on the brain through the right eye	Adult	Measurement of lifespan, climbing assay and mobility assay	Showed the impact of direct brain injury on motor function and survival, offering a model for assessing brain injury responses	[177]

### Drosophila in developmental biology and cancer

From the studies of Poulson [188] and Lewis [189] on Notch and on the homoeotic genes, respectively, *Drosophila* is shown to have been playing a crucial role in developmental biology since the 1930s. In a variety of settings, Notch mediates connections between cells, and abnormalities in Notch signal transduction can lead to a variety of cancers and other disorders [190]. It gave rise to an entire sector that is presently the focus of several biomedical studies [191]. Lewis first identified the homoeotic genes as having an impact on fly body design, and they have since been found to have a variety of functions in nearly all higher eukaryotes [192]. Once more, many genes with homeobox motifs have important functions in cancer [193]. Nüsslein-Volhard and Wieschaus [194] conducted genome-wide forward genetic screening for patterning anomalies in fly embryos, which resulted in the identification of multiple participants in nearly all important developmental pathways, including Toll signalling BMP/TGFb and Wnt, Hedgehog. It is impossible to exaggerate how important these pathways are to our understanding of cancer, developmental diseases, and human development [195].

Cancer typically initiates as a localized disease, but its impact can extend throughout the entire body. Therefore, comprehensive whole-body models are indispensable for comprehending the mechanisms underlying its pathogenesis and for developing effective drugs with a favourable therapeutic index. Indeed, *Drosophila* has proven its worth as a valuable model for cancer research, offering genetic and pharmacological toolkits that aid in the exploration of cancer mechanisms and the development of therapeutic interventions. Exactly, *Drosophila*‘s forward genetics enables the characterization of phenotypes within or between tissues resulting from naturally occurring mutations. Conversely, its reverse genetics enables the modelling of genetic alterations observed in patients, facilitating the exploration of drug responses at the animal level tailored to specific genotypes. This dual approach provides a powerful platform for cancer research and drug discovery.

Early studies dating back to the 1930s identified mutant *Drosophila* strains with mutations in the lethal giant larvae (lgl) gene, which exhibited significant disorganization and hyperproliferation of larval tissues, including the brain and imaginal discs [196]. Upon being transplanted into hosts with a wild-type genotype, cells carrying mutations in the lgl gene demonstrated invasive tendencies, successfully colonizing their local environment [196]. Subsequent genetic investigations in *Drosophila* identified dlg and scrib as interactors with lgl, collectively regulating cell polarity. Loss of cell polarity is observed in approximately 80% of human cancers [197]. Indeed, expression levels of the human orthologs of dlg and scrib are notably lower in various types of cancer compared to their expression levels in normal tissues, as demonstrated by studies conducted by Pearson et al. in 2011 and Sonoshita and Cagan in [198]. Taken together, these findings indicate a functional conservation of dlg, lgl, and scrib as tumour suppressors across different species.

Moreover, studies in *Drosophila* have revealed a phenomenon known as ‘cell competition’, which serves to eliminate cells possessing distinct characteristics. The initial instance was observed in flies harbouring the Minute (M) mutation, affecting a ribosomal gene. Genetic manipulations inducing clones with Minute (M) heterozygosity within wild-type wing discs, characterized by epithelial monolayers, resulted in apoptosis, effectively eliminating these clones while maintaining normal wing size and shape, as demonstrated by studies conducted by Morata and Ripoll in 1975 and Moreno et al. in [199]. Interestingly, genes associated with cancer also play significant roles in cell competition. In the context of cell competition, a cell that overexpresses Myc, termed a ‘supercompetitor’, has been observed to eliminate surrounding wild-type cells in developing wings, as reported in studies by [270] and Moreno et al. in [200]. Likewise, supercompetition arises from a range of genetic abnormalities affecting pathways such as WNT/Wg, Hippo, and JAK-STAT. This suggests that supercompetitors may function as seeds for tumours, as indicated by studies by Tyler et al. in [201] Vincent et al. in [202] and Rodrigues et al. in [203]. Conversely, cell competition plays an anti-tumour role in various contexts. Certain lgl mutant alleles induce proliferation rather than cell death. Examples include a cleaned-up allele of lgl4 or alleles such as lglE2S31, lglE6S, lgl27S3, or lgl23S9, as documented in studies by [271] and 2010. These observations suggest that lgl alleles can lead to distinct phenotypes. In addition to genetic alterations, environmental factors also influence cell competition. For instance, systemic hyperinsulinemia has been shown to disrupt the elimination of scrib mutant cells and promote tumorigenesis in *Drosophila*, as reported by Sanaki et al. in [204]. Similar to *Drosophila*, mammals also engage in cell competition. For example, in cultured non-transformed epithelial monolayers, there is a tendency to exclude a small population of cells expressing oncogenic RAS or SRC from the apical region, as demonstrated by studies conducted by [272] and Kajita et al. in [205]. Similarly, in mice, normal tissues have been observed to eliminate cells exhibiting decline in the level of expression of Myc genes encoding a ribosomal protein, a cell polarity regulator, or components of the Hippo pathway, as evidenced by studies conducted by Norman et al. in [206] Clavería et al. in [207] and Hashimoto and Sasaki in [208]. These findings raise an intriguing possibility that cell competition functions as an intrinsic mechanism to prevent carcinogenesis.

Reverse genetics has enabled the establishment of *Drosophila* models for cancer genotypes. One of the oldest and simplest methods for artificially inducing transgenes is by employing a heat shock promoter, which involves placing transgenic flies in a warm incubator, as described by Ashburner and Bonner in [209]. However, heat shock-induced transgene expression occurs throughout the body, which can lead to developmental abnormalities. Furthermore, there is also a leakage of transgene expression even in the absence of heat shock, as reported by Brand and Perrimon in [38]. In complement to this method, the GAL4/UAS system has proven to be a valuable tool, as demonstrated by Brand and Perrimon in [38] (Figure 2). In essence, this method utilizes the yeast transcription factor GAL4, which is controlled by cell type- or tissue-specific enhancer/promoters, along with its target UAS integrated into the fly genome. This arrangement enables spatial and/or temporal regulation of transgene expression, as outlined by Brand and Perrimon in [38].

#### Drosophila model of colorectal cancer

Colorectal cancer (CRC) stands as the third most frequently diagnosed cancer in both genders worldwide, with approximately 1.8 million new cases and 880,000 deaths reported in 2018. This positions CRC as the second most fatal cancer type on a global scale [210]. Colorectal cancer (CRC) exhibits assortments of genetic irregularities involving RAS oncogenes (NRAS/KRAS/HRAS) and/or tumour suppressor genes such as APC, SMAD4, TP53, and LLGL1 [211]. To comprehend the impact of such diversities on colorectal cancer (CRC) development, genetically engineered mouse models (GEMMs) for intestinal tumours have made crucial contributions [212]. These models have led to the discovery of CRC mechanisms, including the tumour-promoting NOTCH-ABL-TRIO-RHO pathway as well as the PGE2-EP2 pathways, the invasion and metastasis-suppressing Aes gene, which inhibits NOTCH signalling [213–215]. Regrettably, GEMMs with complex genotypes demand significant efforts for their generation and maintenance [216]. In this context, *Drosophila* colorectal cancer (CRC) models have proven to be complementary to mammalian models, offering a rapid platform for scrutinizing the complexity of CRC, including disease mechanisms and drug responses. To model colorectal cancer (CRC) genotypes in flies, Bangi et al. utilized the byn-GAL4 driver, which is active in the hindgut and corresponds to the human colon [217], along with patient genomic data from The Cancer Genome Atlas (TCGA) [218]. Combining active rasG12V expression with RNA interference (RNAi) knockdown of tumour suppressors apc, p53, smad4, and/or PTEN in *Drosophila* recapitulated key colorectal cancer (CRC) pathologies, including cell proliferation, epithelial-mesenchymal transition (EMT), and distant metastasis. Among these combinations, p53RNAi, rasG12V, apcRNAi, ptenRNAi, and induced the most severe phenotypes. Additionally, each fly line exhibited unique responses to anti-cancer agents, underscoring the significance of personalized medicine tailored to individual patient genotypes [218]. In addition to investigating the intricacies of cancer, *Drosophila* also provides a rapid platform for validating hypotheses derived from epidemiological studies. A recent study showcased an association between social isolation and an elevated risk of cancer-related mortality. Additionally, rats subjected to lifelong isolation, spanning up to 18 months, exhibited the development of mammary tumours [219]. Interestingly, in *Drosophila* as well, social isolation was found to accelerate the progression of gut tumours within a span of 21 days [220], underscoring their utility in investigations of risk factors that necessitate long-term observation, a task often challenging when employing mammalian models.

#### Drosophila model thyroid cancer

The incidence of Thyroid cancer (TC) is rising significantly on a global scale. In the United States, projections indicate that by 2030, TC is expected to become the fourth most prevalent type of cancer, supplanting colorectal cancer (CRC), thereby representing one of the most urgent health concerns [221]. Thyroid cancer encompasses subtypes such as papillary thyroid cancer (PTC) and the relatively uncommon medullary thyroid cancer (MTC). An activated form of the cell surface receptor tyrosine kinase (RTK) RET is accountable for approximately 90% of MTC cases. However, progress in drug discovery for MTC treatment has been sluggish, primarily due to the absence of an efficient research platform. To address this challenge, transgenic *Drosophila* models for MTC were developed by inducing the expression of an active M955T isoform of *Drosophila* Ret (dRetM955T) in epithelial tissues, such as the eyes and wing discs. This model mimics the RETM918T mutation found in MTC patients [222–224]. These models were instrumental in validating the lead chemical ZD6474, which led to the development of vandetanib as the first targeted therapy for MTC [225]. Moreover, these models enabled intensive chemical genetic screening, resulting in the successful identification of novel lead compounds that exhibited significantly improved efficacy compared to sorafenib, the Food and Drug Administration (FDA)-approved multikinase inhibitor drug [224]. In contrast to MTC, PTC constitutes approximately 85% of all thyroid cancer cases [226]. PTC exhibits subtypes with distinct genetic profiles for effectors in the RTK-MAPK pathway, including oncogenic RET fusion genes found in 30% of PTC patients [227]. While RET inhibitors demonstrate effectiveness in this cohort, they also induce severe toxicity [228]. Among the identified CCDC6-RET and NCOA4-RET fusions, the latter leads to more severe pathogenesis in patients, with mechanisms and therapeutic options yet to be determined [229]. Similar to MTC, *Drosophila* emerged as a potent tool in addressing this cancer. Specifically, flies expressing CCDC6-RET or NCOA4-RET driven by the patched (ptc) promoter exhibited enhanced migration, delamination, and epithelial-mesenchymal transition (EMT) of transformed cells [227]. In these fly models, the patched (ptc) promoter directs the expression of transgenes in developing epithelial tissues, including eye, wing, leg discs, as well as other tissues [222]. Comprehensive kinome screening revealed that NCOA4-RET signalled through kinases, such as WEE1, which were distinct from those associated with CCDC6-RET. Inhibiting the NCOA4-RET-WEE1 network through the synergistic action of sorafenib and the WEE1 inhibitor AZD1775 effectively suppressed the aforementioned phenotypes, presenting a novel candidate therapy for NCOA4-RET-positive PTC [227].

#### Drosophila model of lung cancer

Throughout the world, lung cancer has consistently maintained the highest mortality rate among all cancer types, with non-small cell lung cancer (NSCLC) representing approximately 85% of all diagnosed cases [230]. Being the most frequently mutated oncogene in NSCLC, KRAS confers resistance to adjuvant chemotherapy and EGFR inhibitors [231]. To identify potential therapeutic candidates for KRAS-positive NSCLC, *Drosophila* served as a testing ground, utilizing its tracheal system, which develops analogously to the vertebrate lung. The breathless (btl)-GAL4 driver was utilized to target the misexpression of *Drosophila* rasG12V and knockdown of PTEN specifically to the trachea in *Drosophila*. This resulted in the development of tumour-like growths and lethality in early larval stages (Levine Benjamin et al., 2016). After conducting chemical screening of a library containing 1192 FDA-approved drugs, trametinib, a MEK inhibitor, and fluvastatin, an HMG-CoA reductase inhibitor, emerged as potential candidates to formulate a therapeutic cocktail. Indeed, they synergistically curtailed the growth of A549 human NSCLC cells harbouring active KRASG12S55. *Drosophila* has also contributed to the development novel therapeutic approaches for individuals harbouring the KIF5B-RET fusion oncogene, which is the most significant fusion driver in NSCLC [232]. Specifically, the product of KIF5B-RET activated multiple RTKs, including EGFR, providing vulnerabilities that could be targeted using combinations such as sorafenib with erlotinib or paclitaxel as potential treatment options for KIF5B-RET-positive non-small cell lung cancer (NSCLC). These therapies are awaiting validation in patients [232].

#### Drosophila model of brain tumor

Gliomas represent the most prevalent intracerebral tumours, with glioblastoma multiforme (GBM) being the most aggressive among them, characterized by limited effective therapies and a median patient survival of only 15 months. While studies employing genetically GEMMs have shed light on the mechanisms underlying GBM development and therapeutic resistance, the development of novel therapeutic strategies has remained exceedingly challenging for decades [233]. To address this challenge, Read et al. pioneered the creation of *Drosophila* models mimicking GBM genotypes by introducing activated isoforms of *Drosophila* Egfr (dEGFRλ) and p110 (dp110CAAX) using the glia-specific repo-GAL4 driver [234]. The introduction of these transgenes induced infiltration and glial proliferation, as well as loss of cell polarity, mirroring the characteristics of human glioma and resulting in larval lethality [234]. These observed phenotypes were found to be reliant on TOR, CCNG1-CDKs, MYC, as well as RB-E2F pathways, indicating them as potential novel targets for GBM therapy [234]. Consequently, *Drosophila* provides a practical platform for elucidating signalling networks involved in cancer development.

### Drosophila models of metabolic and hepatic diseases

The study of metabolic and hepatic diseases using *Drosophila melanogaster* has significantly contributed to our understanding of these complex pathologies. Although *Drosophila* lacks a liver, its fat body (as shown in Figure 3) serves a similar function by regulating energy storage, metabolism and immune responses. This section explores how *Drosophila* models have advanced the study of metabolic diseases, particularly those related to hepatic function.

#### Linking fat body to liver function

The *Drosophila* fat body is a multifunctional organ that plays a central role in lipid and carbohydrate metabolism, much like the mammalian liver. It is involved in the storage and mobilization of energy reserves, as well as in detoxification processes. Due to its analogous functions, the fat body is a valuable model for studying metabolic disorders such as obesity, diabetes and fatty liver disease (steatosis), which are characterized by lipid dysregulation and insulin resistance [41].

#### Modeling hepatic metabolic disorders in Drosophila

*Drosophila* has been used extensively to model aspects of metabolic syndrome, a cluster of conditions that includes obesity, insulin resistance and non-alcoholic fatty liver disease (NAFLD). Researchers have employed *Drosophila* to study the genetic and environmental factors that contribute to these conditions, leveraging the genetic tools available in flies to dissect the pathways involved in lipid metabolism and insulin signalling.

For instance, high-sugar and high-fat diets in *Drosophila* lead to the development of obesity-like phenotypes, including increased fat storage in the fat body, insulin resistance and reduced lifespan – paralleling human metabolic disorders (Palanker [235]. These models have provided insights into the molecular mechanisms driving these diseases, such as the role of key regulatory genes like foxo and slif, which are involved in insulin signalling and lipid metabolism.

Moreover, *Drosophila* has been instrumental in studying the pathogenesis of NAFLD, a common hepatic manifestation of metabolic syndrome. Flies fed a high-fat diet exhibit lipid accumulation in the fat body, akin to hepatic steatosis in humans. This model has been used to identify genetic modifiers of fat storage and to explore the interplay between diet, lipid metabolism and inflammation – key factors in the progression from steatosis to more severe liver diseases [236].

#### Contributions to understanding metabolic and hepatic pathologies

*Drosophila* models have also contributed to the discovery of potential therapeutic targets for metabolic diseases. For example, studies in flies have identified the role of lipophorin receptors in lipid transport and their regulation by insulin signalling, providing potential avenues for therapeutic intervention in conditions like hyperlipidaemia and atherosclerosis [237]. Additionally, genetic screens in *Drosophila* have uncovered novel genes that regulate lipid storage and glucose metabolism, offering new insights into the genetic basis of metabolic diseases.

### Drosophila in the modeling of cardiac and muscular diseases

The utility of *Drosophila melanogaster* as a model organism extends into the study of cardiac and muscular diseases, offering insights into the molecular and genetic mechanisms underlying these conditions. Despite significant anatomical differences between *Drosophila* and humans, key aspects of cardiac and muscle physiology are conserved, making *Drosophila* a valuable model for studying heart function, muscle development and the genetic causes of cardiomyopathies and muscular dystrophies.

#### Cardiac disease models in Drosophila

The *Drosophila* heart, also known as the dorsal vessel, is a simple tubular structure composed of contractile cardiomyocytes. While structurally simpler than the human heart, it shares many conserved molecular pathways with the mammalian cardiovascular system, including those involved in cardiac development, contractility and ageing [238]. These similarities make *Drosophila* an effective model for studying both congenital heart defects and age-related cardiac dysfunction.

##### Congenital cardiomyopathies

Mutations in genes encoding structural proteins and ion channels are often responsible for congenital cardiomyopathies in humans. *Drosophila* has been used to model these diseases by introducing mutations in orthologous genes. For instance, mutations in dSUR, the *Drosophila* ortholog of the human SUR gene, which encodes a subunit of the ATP-sensitive potassium (KATP) channel, have been shown to cause defects in cardiac excitability and contractility [239]. These models provide insight into the molecular mechanisms underlying ion channelopathies and help identify potential therapeutic targets.

##### Cardiac aging and heart failure

*Drosophila* models have also been extensively used to study age-related cardiac dysfunction. As flies age, they exhibit declines in cardiac performance, including arrhythmias, reduced contractility, and increased heart failure, which are similar to the ageing-related changes seen in the human heart. These phenotypes are exacerbated by high-fat diets, which cause lipid accumulation and further deterioration of heart function [240]. Through the use of genetic screens, researchers have identified genes involved in lipid metabolism, oxidative stress, and mitochondrial function that contribute to cardiac ageing and failure, providing potential avenues for therapeutic intervention.

#### Muscular disease models in Drosophila

*Drosophila* has also proven to be an effective model for studying muscular diseases, including muscular dystrophies and myopathies. The fly’s musculature consists of somatic muscles, which are functionally analogous to vertebrate skeletal muscles, and indirect flight muscles, which serve as models for studying muscle integrity, structure, and function [241].

##### Muscular dystrophies

Muscular dystrophies are a group of genetic disorders characterized by progressive muscle weakness and degeneration. *Drosophila* models of muscular dystrophy have been generated by introducing mutations in genes that are orthologous to those associated with human dystrophies. One of the best-characterized models is *Drosophila* Duchenne muscular dystrophy (DMD), caused by mutations in the Dys gene, the fly homolog of the human DMD gene. Flies with Dys mutations exhibit progressive muscle degeneration, impaired locomotion, and shortened lifespan, closely mimicking the symptoms of DMD in humans [242].

These models have been instrumental in identifying the molecular pathways involved in muscle degeneration and in testing potential therapies. For example, studies in *Drosophila* have shown that enhancing autophagy, a process of cellular self-degradation, can mitigate muscle degeneration in DMD models [243]. These findings have important implications for the development of treatments aimed at preserving muscle function in dystrophic patients.

##### Congenital myopathies

*Drosophila* has also been used to model congenital myopathies, a group of muscle disorders characterized by structural abnormalities in muscle fibres. Mutations in genes encoding proteins involved in muscle structure, such as Actn (encoding alpha-actinin) and Kettin, have been shown to cause muscle defects in *Drosophila* that are similar to those observed in human congenital myopathies [244]. These models allow researchers to study the genetic and molecular basis of muscle fibre organization and to explore therapeutic strategies that target these pathways.

#### Contributions to understanding cardiac and muscular diseases

The use of *Drosophila* in cardiac and muscular disease research has led to several important discoveries, particularly regarding the genetic and molecular pathways that regulate heart and muscle function. These models have provided insight into the role of ion channels, cytoskeletal proteins, and metabolic pathways in maintaining the structural and functional integrity of the heart and muscles. Moreover, *Drosophila* models have been used to test potential therapeutic interventions, including small molecules, gene therapies and dietary modifications, that may alleviate symptoms and slow disease progression in patients with cardiac or muscular disorders.

### Drosophila model of infectious diseases

*Drosophila* has emerged as a powerful model for studying infectious diseases, particularly in understanding host-pathogen interactions and the immune response. The fly’s innate immune system shares many similarities with the human immune system, including the Toll and Imd pathways, which play crucial roles in defence against bacterial and fungal infections [245]. These pathways have been extensively studied in *Drosophila* to elucidate the molecular mechanisms of immune signalling and pathogen recognition. In bacterial infection models, *Drosophila* has been used to study the pathogenesis of various human pathogens, including *Pseudomonas aeruginosa*, *Mycobacterium tuberculosis* and *Staphylococcus aureus* [246]. These models have provided insights into the virulence factors of these pathogens and the host’s immune response, leading to the identification of novel antimicrobial targets.

*Drosophila* has also been utilized to model viral infections, including those caused by human pathogens such as influenza and dengue virus. The fly’s ability to mount an antiviral response through the activation of RNA interference (RNAi) and other immune pathways has been pivotal in understanding the genetic factors that contribute to viral susceptibility and resistance [247]. These studies have implications for the development of antiviral therapies and for understanding the host-pathogen co-evolution. In the context of parasitic infections, *Drosophila* models have been used to study the interaction between the host and parasitic organisms, such as Plasmodium species, the causative agents of malaria. While *Drosophila* is not a natural host for Plasmodium, transgenic approaches have enabled the expression of Plasmodium genes in *Drosophila*, allowing for the study of parasite biology and host immune responses in a genetically tractable system [248].

### Drosophila in drug screening and toxicological studies

*Drosophila melanogaster* has become an invaluable tool in drug discovery and toxicology due to its genetic tractability, short life cycle and the availability of high-throughput screening techniques. This section discusses the dual role of *Drosophila* in drug screening and toxicological studies, highlighting how this model organism contributes to identifying new therapeutics and assessing drug safety.

#### Drosophila in drug screening

The use of *Drosophila* in drug screening is rooted in its capacity to model complex human diseases and to evaluate the efficacy of potential therapeutics in a whole-organism context. The genetic similarities between *Drosophila* and humans allow for the testing of drugs that target conserved pathways, providing insights into their therapeutic potential and mechanisms of action.

One of the key advantages of using *Drosophila* in drug screening is the ability to perform high-throughput genetic and pharmacological screens. For example, *Drosophila* models of neurodegenerative diseases, such as Alzheimer’s and Parkinson’s, have been used to screen libraries of small molecules for compounds that can ameliorate disease symptoms, such as improving motor function or reducing neurodegeneration [249]. These screens have led to the identification of potential therapeutic compounds that can be further validated in mammalian models and clinical trials.

Additionally, *Drosophila* has been employed in screens to identify drugs that target metabolic pathways. For instance, in models of obesity and diabetes, flies can be treated with various compounds to assess their effects on lipid metabolism, insulin sensitivity and glucose homoeostasis [240]. The ability to screen large numbers of compounds in a cost-effective and time-efficient manner makes *Drosophila* a valuable asset in the early stages of drug development.

#### Drosophila in toxicological studies

In toxicology, *Drosophila* provides a robust platform for assessing the safety and potential side effects of drugs and environmental toxins. The genetic and physiological conservation between *Drosophila* and humans means that many toxicological responses observed in flies are relevant to human health.

Toxicological studies in *Drosophila* typically involve exposing flies to various compounds and assessing their effects on survival, reproduction, development and behaviour. For example, *Drosophila* has been used to study the toxic effects of environmental pollutants, such as pesticides and heavy metals, on neurological function and development [250]. These studies have provided critical data on the potential risks associated with exposure to these substances, contributing to the development of safer chemical compounds and regulatory policies.

Moreover, *Drosophila* is well-suited for studying the mechanisms of drug toxicity. By utilizing genetic tools, such as the GAL4/UAS system, researchers can overexpress or knock down genes involved in drug metabolism and detoxification, allowing for the identification of pathways that mediate drug-induced toxicity. For example, *Drosophila* models have been used to study the hepatotoxic effects of acetaminophen, revealing the role of cytochrome P450 enzymes in drug metabolism and the generation of toxic metabolites [251].

The integration of high-throughput screening with toxicological assessment in *Drosophila* provides a comprehensive approach to drug development. It allows researchers to not only identify promising therapeutic candidates but also to evaluate their safety profiles early in the drug development process, reducing the risk of adverse effects in later stages of testing.

#### Contributions to drug discovery and toxicology

The contributions of *Drosophila* to drug discovery and toxicology extend beyond the identification of therapeutic candidates. This model organism has also been instrumental in elucidating the molecular mechanisms underlying drug action and toxicity, offering insights that are directly translatable to human health. The combination of genetic tools, whole-organism screening and the ability to model human diseases makes *Drosophila* a powerful system for advancing drug discovery and ensuring the safety of new therapeutics.

## Crispr-CAS9 system in Drosophila research: revolutionizing disease modeling

The CRISPR-Cas9 system has emerged as a groundbreaking tool in genetic research, enabling precise genome editing with unprecedented efficiency and accuracy. In *Drosophila melanogaster*, CRISPR-Cas9 has not only expanded the toolkit available to geneticists but also fundamentally changed the way disease models are generated and studied.

The CRISPR-Cas9 system functions by using a guide RNA (gRNA) to target specific DNA sequences within the genome. The Cas9 enzyme, guided by the gRNA, introduces double-stranded breaks at the targeted site. These breaks can then be repaired by the cell’s natural DNA repair mechanisms – either through non-homologous end joining (NHEJ), which often results in small insertions or deletions (indels), or homologous recombination (HR), which can be used to introduce specific mutations or foreign DNA sequences into the genome [42].

The application of CRISPR-Cas9 in *Drosophila* research has revolutionized disease modelling in several significant ways:

### Introduction of human disease mutations

Before the advent of CRISPR-Cas9, introducing specific mutations associated with human diseases into *Drosophila* was challenging, often relying on random mutagenesis or less precise techniques like homologous recombination with low efficiency. CRISPR-Cas9 has made it possible to introduce precise point mutations, deletions, or insertions into *Drosophila* genes that are orthologous to human disease genes. This capability allows researchers to replicate human disease mutations in *Drosophila* with high fidelity, creating more accurate models of diseases such as cancer, neurodegenerative disorders, and metabolic syndromes [252].

For instance, CRISPR-Cas9 has been used to introduce the same mutations found in human patients with Alzheimer’s disease, Parkinson’s disease, or cancer into the corresponding *Drosophila* genes. These models can then be used to study the molecular mechanisms of disease progression and to test potential therapeutic interventions, providing insights that are directly relevant to human health [43].

### Creation of complex genotypes

CRISPR-Cas9 allows for the generation of complex genotypes by enabling the simultaneous editing of multiple genes or the introduction of large genetic constructs. This capability is particularly useful for studying polygenic diseases or for creating multi-mutant models that better reflect the genetic complexity of human diseases. For example, researchers can now generate *Drosophila* models that carry mutations in multiple genes known to interact in the context of a particular disease, providing a more comprehensive understanding of gene-gene interactions and their impact on disease phenotypes [55,253].

This ability to create complex genotypes also extends to the development of sophisticated genetic tools, such as reporter constructs or gene knock-ins, that can be used to track disease progression or to study the effects of specific mutations in real-time.

### High-throughput screening and functional genomics

The ease and efficiency of CRISPR-Cas9-mediated genome editing have facilitated large-scale genetic screens in *Drosophila*. Researchers can now systematically knock out or modify genes across the *Drosophila* genome to identify those that play critical roles in disease processes. These screens can be conducted in a high-throughput manner, allowing for the rapid identification of novel disease-related genes or potential drug targets [254].

High-throughput CRISPR screens are particularly valuable in functional genomics, where the goal is to understand the roles of all genes within a particular pathway or network. By systematically perturbing genes across the genome, researchers can uncover new interactions and pathways that contribute to disease, opening up new avenues for therapeutic intervention.

### Precision medicine and personalized disease models

CRISPR-Cas9 is also paving the way for precision medicine in *Drosophila* research. By introducing patient-specific mutations into *Drosophila*, researchers can create personalized disease models that reflect the genetic makeup of individual patients. These models can be used to study the specific effects of these mutations and to test personalized therapeutic strategies, providing a platform for developing tailored treatments that are more effective and have fewer side effects [255].

Personalized *Drosophila* models are particularly valuable for studying rare genetic disorders, where patient-specific mutations can be difficult to model in traditional systems. By replicating these mutations in *Drosophila*, researchers can gain insights into the disease mechanisms at work in individual patients and explore potential therapeutic approaches that are uniquely suited to their genetic profile.

### Future directions and implications

As CRISPR-Cas9 technology continues to evolve, its applications in *Drosophila* research are expected to expand even further. The development of more refined CRISPR techniques, such as base editing or prime editing, will allow for even more precise modifications to the *Drosophila* genome, reducing off-target effects and increasing the fidelity of genetic edits [256]. These advancements will enable researchers to model diseases with unprecedented accuracy, leading to deeper insights into disease mechanisms and more effective therapeutic strategies.

Moreover, the integration of CRISPR-Cas9 with other emerging technologies, such as single-cell sequencing and advanced imaging techniques, will provide new opportunities for studying disease processes at the cellular and molecular levels. These integrated approaches will allow for a more comprehensive understanding of how genetic mutations drive disease phenotypes and how these processes can be targeted therapeutically.

## Investigating non-conserved genes and mechanisms to advance public health

Some of the biggest risks to human health are vector-borne illnesses. Due to their role as carriers of several common infectious diseases, mosquitoes are often time regarded as the deadliest organism on earth [257]. Although much of our knowledge of the genetics of insects comes from research on flies, tactics for controlling mosquito populations can benefit from the biology, genetics, and technological advancements of the *Drosophila* genus. Insecticides, for instance, are one of the first lines of protection against diseases carried by vectors. Numerous of these compounds influence the insect neural system’s channels, receptors, and enzymes; some of these have been thoroughly investigated in the *Drosophila* [258]. Lately, *Drosophila* populations have been used to identify the molecular pathways behind insecticide resistance [259]. In order to generate a list of prospective targets for novel pesticides, research pertaining genes encoding proteins of the nervous system of insect will be necessary, irrespective of whether it is a conserved gene or not. Certain insecticides work by affecting proteins which are essential for the growth of insects but redundant or nonexistent in mammals, and other animals. These include substances that have been extensively researched in *Drosophila*, such as chitin enzyme-producing inhibitors [260,261]). Studies of *Drosophila* genes that lack evident direct human homologs are nonetheless crucial, given that a portion of non-conserved genes are also necessary for survival in these flies [262].

Research into insect-specific biological processes and phenomena may also provide fresh approaches and tools in the fight against vector-borne diseases. Wolbachia are bacterial species that infect many insect and animal species [263]. Wolbachia are vertically transmissible and have a variety of effects on an animal’s fitness. Although Wolbachia was initially found in mosquitoes [264], studies on *Drosophila* made it easier to examine these microbes and demonstrated how they affect the host’s ability to reproduce and lifespan [265]. A Wolbachia strain, known as wMel, can quickly spread among mosquitoes and can also stop dengue virus from being transmitted. In fact, mosquitoes carrying the wMel virus were released at two field sites in Australia and quickly spread to the native population [266]. While this investigation is still being carried out to track the real dengue fever control in the region, it serves as a great illustration of how to ‘translate’ *Drosophila* research findings to advance public health.

Advances in *Drosophila* genome engineering have made it possible to modify the genomes of other insect species [267]. Transgenic vectors can be created using mosquito molecular techniques to stop the spread of infection [268]. One example is the creation of transgenic Anopheles mosquitoes with increased resistance to the Plasmodium malaria pathogen [269]. Therefore, advancements in *Drosophila* technology will continually offer researchers practical tools for studying and modifying the genomes of disease vectors in other insect species.

**Table:** Public databases that support research on human genes that are similar to Drosophila genes

Resource	Illustration	Features	Universal Resource Locator (URL)
1000 Genomes Project	Individuals from several populations were sequenced with lower coverage, but phenotypic information was lacking.	Most variants can be found by gene or browser searches and are more prevalent in populations.	www.1000genomes.org
Entrez Gene	Summary from NCBI for each gene	Examples and resources for pathogenic alleles are provided by links to CliniVar and dbVar.	www.ncbi.nlm.nih.gov/gene
DECIPHER	Database of structural variations from willing subjects displaying developmental characteristics	Gene or region searches are also available, as well as phenotype-linked tracks within the UCSC genome browser.	www.decipher.sanger.ac.uk
Human Gene Mutation Database (HGMD)	It gathers information on known, published human mutations that cause diseases	It requires non-profit organization registration. It gives information and references on alleles known to cause disease	www.hgmd.cf.ac.uk
Genotype and phenotypic database	Data with restricted access that includes deidentified subjects’ genotypes and phenotypes from many research	Requires the NIH Data Access Committee to allow access before reviewing sequencing data	www.ncbi.nlm.nih.gov/gap
ClinVar	Researcher-submitted, diagnostic-lab-submitted, and other sources submitted genetic variation and associated phenotypes archive	A gene’s variation can be filtered depending on the type of variation, the molecular impact, and the number of researchers that have submitted their findings to uncover a deleterious mutation in the gene of interest.	www.ncbi.nlm.nih.gov/clinvar

## Conclusions

*Drosophila melanogaster* remains a cornerstone of biological research, offering unparalleled versatility and insights into fundamental biological processes. Its genetic diversity, rapid generation time, and affordability make it an invaluable model organism for studying a wide range of human diseases, including neurodegenerative disorders and cancer. Recent advancements in genetic engineering, such as CRISPR-Cas, have further expanded its utility, enabling precise genome editing and tailored disease modelling. *Drosophila* serves as a platform for understanding molecular mechanisms underlying immunity, tissue regeneration/degeneration, and environmental stress responses. As we continue to explore the intricacies of genetics and disease, *Drosophila* stands as a powerful tool for driving innovation and discovery, ultimately contributing to advancements in biomedicine and human health.

## Data Availability

Data available on request from the corresponding author.
